# A System dynamics analysis of the factors influencing the promotion of prefabricated decoration, based on a co-occurrence network

**DOI:** 10.1371/journal.pone.0331703

**Published:** 2025-09-04

**Authors:** Yingchen Wang, Jingtian Liu, Guiwei Zhang, Xiaoxiao Geng, Yan Liu

**Affiliations:** 1 School of Management Engineering and Business, Hebei University of Engineering, Handan, Hebei Province, China; 2 School of Architecture and Art, Hebei University of Engineering, Handan, Hebei Province, China; SASTRA Deemed University, INDIA

## Abstract

Prefabricated decoration effectively addresses resource waste, environmental pollution, and quality issues associated with traditional decoration methods. It also enhances the functionality of prefabricated buildings and offers promising market potential. Currently, insufficient attention from both the public and managers hinders prefabricated decoration development. To address this, this study analyzed 68 relevant documents from the China National Knowledge Infrastructure and Web of Science databases, identified 34 factors affecting the promotion of prefabricated decoration, and constructed a co-occurrence network model based on an adjacency matrix. The centrality index was used to identify four key factors related to the three core stakeholders: government support, technical level, developers’ willingness to use, and consumers’ willingness to use. Subsequently, the four key factors were simulated using Vensim software. The evolutionary trends of the system and the interactions between these factors were analyzed. The findings reveal that both developers and consumers show strong sensitivity to financial subsidies. As a result, the government can effectively increase subsidies to encourage adoption of prefabricated decoration. Additionally, enhancing the use of BIM technology throughout the project life cycle has been shown to promote synergy within the industrial chain, thereby improving promotion efficiency. Consumer feedback is essential for ensuring policy accuracy and plays a crucial role in advancing the industry's development. The novel contributions of this paper include the integration of co-occurrence network analysis with a system dynamics model, identifying key factors through the co-occurrence network, and uncovering the complex interactions between these factors via the system dynamics model. Based on the simulation results, this study predicts the development prospects of prefabricated decoration, offering both a theoretical foundation and practical guidance for policy formulation and promotion strategies.

## 1. Introduction

With the promotion of green development policies, issues like environmental pollution, resource waste, and poor quality in traditional decoration methods have worsened. These problems are global. Prefabricated buildings, compared to traditional and green buildings, offer better quality, efficiency, and sustainability [1]. They help reduce environmental impact [2] and improve urban building environment [3]. In addition, the prefabricated mode can mitigate the problems associated with the long construction periods of traditional buildings to some extent, such as difficulty guaranteeing construction quality, construction noise pollution, and significant carbon emissions. Consequently, the prefabricated method has become a key alternative to traditional decoration. As a major example of building industrialization, prefabricated buildings include “S” systems (main industrialization), “I” systems (built-in industrialization), and prefabricated landscapes (landscape industrialization) [4]. This paper focuses on the “I” system of prefabricated decoration. The system uses a dry construction method to assemble and install factory-made built-in components on-site [5].

Prefabricated decoration, as a modern building method, has gained widespread attention and developed rapidly in recent years. Through optimisation and innovation, prefabricated building technology has gradually become the mainstream method in decoration projects [6]. This method significantly enhances construction efficiency by utilizing prefabricated components and on-site assembly. It also effectively mitigates environmental pollution and resource waste at construction sites, aligning with the current emphasis on green buildings and sustainable development. Meanwhile, prefabricated decoration is an innovative method for renovating existing buildings, likely driving changes in the interior building industry.

Globally, especially in developing countries, prefabricated decoration technology has emerged as a key direction for the transformation and upgrading of the construction industry. With technological advancements and changing market demands, prefabricated decoration has expanded beyond traditional buildings to emerging fields such as green and intelligent buildings. Research focuses on improving material utilization efficiency, optimizing the construction process, and improving construction quality. Prefabricated decoration has many advantages. However, it still faces several practical challenges. These include selecting appropriate materials during design and construction, determining the optimal construction sequence, and making decisions based on multiple standards. Each of these issues requires further exploration. Compared with other regions, the construction industry in Hebei Province still lags behind [7]. As a key component of the Beijing-Tianjin-Hebei integrated development strategy, Hebei Province must align with Beijing and Tianjin, actively promoting sustainable and environmentally friendly construction and decoration methods. Although prefabricated decoration lags behind prefabricated buildings, its adoption will significantly advance the modernization of Hebei Province's construction industry and contribute to environmental protection and resource conservation. This will stimulate economic growth in Hebei Province and accelerate its overall development. In recent years, Hebei Province has introduced policies to support the advancement of prefabricated decoration. Despite significant advancements in prefabricated decoration technology, challenges remain compared to traditional cast-in-place methods, including limited industrial scale and restrictive policies [8]. In addition, from the demand-side perspective, public awareness of prefabricated decoration remains low. This has become one of the main obstacles to promoting its wider application. From the perspective of the supply side, suppliers’ understanding of prefabricated decoration is not deep. Most enterprises focus on the production of basic components, so lack in-depth knowledge about product innovation, technological upgrading, and market adaptability. This lack of innovation power seriously restricts the development and application of prefabricated decoration technology at a higher level and in a wider range of fields, hindering its widespread popularization.

The existing literature has studied the factors influencing the development of prefabricated decoration, but simply and quantitatively according to weight [9], without systematically analyzing the mechanism of influencing factors. There are also documents that study the importance of government intervention in promoting prefabricated building, without considering other participants [10]. Therefore, the gaps in the existing research are obvious. From the perspective of methodology, it only relies on weight for simple quantitative processing, but does not incorporate visual analysis methods, so a systematic and intuitive process to analyze the influencing factors is lacking. Current literature largely emphasizes the government dimension, neglecting the role of developers and consumers as key stakeholders. This leads to a lack of comprehensiveness in the analysis framework and a failure to produce an overall picture that incorporates the perspectives of multiple subjects. Judging from the research content, most studies focus on the static evaluation of key influencing factors, identifying and weighting them, while rarely addressing the prediction and dynamic analysis of the future development of prefabricated decoration promotion. There is therefore a failure to fully consider potential changes in long-term promotion efficiency and the far-reaching impacts on the overall efficiency of the system. In summary, the above research defects seriously weaken the promotion of prefabricated decoration. This not only increases the cost burden but also delays the process. This has had an important impact on the promotion of prefabricated-type decoration.

The effective promotion of prefabricated decoration is a complex system influenced by factors such as government support, the completeness of the technical system, and decoration costs [11]. Thus, it is essential to systematically clarify the role of key factors in the promotion process and predict their future impact on the development of prefabricated decoration. In this context, the paper innovatively applies a co-occurrence network and system dynamics to promote prefabricated decoration. Many factors affect the popularization of prefabricated decoration, and a co-occurrence network can visualize their complex relationships. This provides a new perspective and a quantitative analysis method for the popularization of prefabricated decoration. The system dynamics method can simulate multiple scenarios and analyze how key factors influence promotion under different policies and environments. This approach helps clarify future trends in prefabricated decoration. In addition, this paper examines how the government, developers, and consumers interact and influence promotion. It also builds a multi-dimensional analysis framework. This provides a new research perspective for promoting prefabricated interior decoration. It also offers a new theoretical basis for policy formulation and promotion strategies.

The remainder of the paper is structured as follows: the literature review section delves into the current state of development of prefabricated decoration, the barriers to its promotion, and the application of Multi-criteria Decision Analysis (MCDA) and system dynamics in this field. The Materials and methods section selects 68 articles from 2015 to 2025 related to “prefabricated decoration,” “promotion,” and “system dynamics” from CNKI and Web of Science. After screening, 34 influencing factors involving 6 major stakeholders are identified, and word frequency analysis is conducted. Based on this, an adjacency matrix is built, and four key factors are identified using the centrality index of a co-occurrence network. A system dynamics model is then established to analyze the mechanisms and verify the model's feasibility. In Results and discussion section, a basic scenario simulation, multi-scenario simulation, and sensitivity testing of the key factors are carried out using Vensim software to analyze the evolution of the key influencing factors. Based on the results of the simulations, targeted policy recommendations for the three major stakeholders--namely, the government, developers, and consumers--are proposed. In Conclusions section, the work is summarized, and the study's limitations and future research directions are outlined.

## 2. Literature review

### 2.1. Development status of prefabricated decoration

Given the continuous improvements in prefabrication technology, various countries are actively promoting prefabricated decoration and related industries. About half of the apartment buildings in Ukraine were built between 1960 and 1980, and they are mainly prefabricated reinforced concrete houses [12]. In the 1960s, the USA, Japan, and other countries began exploring prefabricated interior decoration. They focused on unifying standardization with individualization, modularization with diversification, and functionality with aesthetics [13]. Because prefabrication technology has the potential advantage of creating housing quickly and affordably, developing countries such as India have also begun to rely on prefabrication [14]. As early as 2015, the Hong Kong government introduced an incentive scheme. It offered floor area concessions to private developers to encourage the use of prefabricated technology [15]. In Sweden, prefabricated buildings account for over 80% of the housing market. [16]. Most American houses are low-rise wooden houses built in the suburbs. They applied advanced aviation manufacturing technology to housing in a groundbreaking way [17]. Japanese residential building design generally includes interior decoration, moving from civil engineering to decoration in one step [18]. Germany and Poland recognized the potential of innovative reinforced concrete precast technology through systematic design, construction processes, and the use of Building Information Modeling (BIM). They actively promoted its adoption [19]. The Malaysian government has taken various measures to improve the performance and quality of local projects, including the adoption of prefabricated buildings [20]. Many countries, including Singapore, are using prefabricated components to construct high-rise buildings in both the private and public sectors [21].

The Central Committee of the Communist Party of China and local governments have issued various policies and regulations, including national and local bills, to strongly promote the development of prefabricated decoration. In 2016, the Guiding Opinions of the General Office of the State Council on Vigorously Developing Prefabricated Buildings (Guo Ban Fa [2016] No. 71) proposed several measures. These include improving standards and specifications, innovating prefabricated building design, optimizing component production, enhancing construction quality, and promoting full renovation of buildings [22]. Since the implementation of the “Evaluation Standard for prefabricated Buildings” in 2018, this efficient and sustainable decoration method has been strongly encouraged [23]. In January 2022, the Ministry of Housing and Urban-Rural Development released the “Construction Industry Development Plan of the 14th Five-Year Plan”. It called for vigorous development of prefabricated buildings and the active promotion of prefabricated decoration methods [24]. The Hangzhou Building Industrialization Office issued the Implementation Plan of Hangzhou Prefabricated Decoration Pilot Work (referred to as the “Implementation Plan”). It focuses on green development, promotes the construction of prefabricated decoration systems, and supports the adjustment, transformation, and upgrading of the construction industry's industrial structure [25]. The Jiangxi Provincial Department of Housing and Urban – Rural Development, in collaboration with the Jiangxi Provincial Development and Reform Commission and the Jiangxi Provincial Market Supervision Administration, issued the Guiding Opinions on Strengthening the Construction and Management of Fully Decorated Finished Houses (Gan Jian Zi [2021] No.10). The policy aims to improve housing quality, reduce resource waste and environmental pollution from decentralized decoration, and enhance the living environment [26].

### 2.2. Research on obstacles to prefabricated decoration

Many scholars have examined the factors influencing the development of prefabricated decoration. Zhang et al. used structural equation modeling to show that policy factors play a dominant role, with management and market factors also being key influences [27]. Lu et al. have developed an analytical framework to seek the best level of prefabrication under a certain PEST (political, economic, social, and technical) background. The framework contains 13 PEST factors that affect the adoption of prefabrication. The authors believe that factors such as policy, supply, labor, social attitude, and user acceptance will affect the use of prefabrication technology [28]. Wu built a five-cluster model and concluded that technology lock-in is the most important factor. In addition, business expansion, improved project delivery quality, investment in technological innovation, and returns on technology investments are key influencing factors [29]. Janappriya et al. studied Sri Lanka and found several major obstacles. These include difficulties in transporting prefabricated components, high capital investment costs, and limited understanding of prefabricated construction benefits among owners and developers [30].

Li et al. analyzed news reports retrieved from the China National Knowledge Infrastructure (CNKI) and nine other major portals in China. They concluded that transportation cost was the main obstacle [31]. Zhong and other researchers used the Analytic Hierarchy Process (AHP) to construct a cost evaluation system. The study found that design phase costs, apartment modifications, and installation labour costs had the greatest impact on renovation costs [32]. However, Jiang et al. argue that risk across the entire industrial chain—including design, prefabrication, and operation—is a more significant obstacle than cost [33]. Ouyang addressed the problems of high cost, insufficient design coordination, and a shortage of industrial workers by suggesting intelligent construction [34]. In addition, Zeng et al. proposed measures to standardize the selection of prefabricated building decoration. They also identified ongoing issues in China, including the absence of a unified standardization system and inconsistent quality control standards [35]. Zhang et al. analyzed the main influencing factors, characteristics, development needs, and implementation strategies of prefabricated residential decoration. They found that decoration design and construction are not well integrated, and the number of decoration components remains limited [36]. Hou et al. applied a DEMATEL-ISM model to analyze the hierarchical structure and causal relationships among various factors. They built a multi-layer hierarchical model. The results showed that policy standards and technological innovation are the most critical factors. Construction environment, management environment, and operation specifications are mid-level factors. Material quality, acceptance, and equipment selection are surface-level factors. Cai et al. used the multiplication method MICMAC to explain the ISM structural model and applied a cross-influence matrix to identify the five-level structural relationships, along with the dependence and driving force of each factor. The results show that the prefabrication rate is the root factor influencing the construction and promotion of prefabricated buildings in Hangzhou [37].

### 2.3. Application of multi-criteria decision making (MCDA) in the field of prefabricated decoration

Multi-criteria decision making (MCDA) is a method used to support decision-making when multiple criteria or guidelines need to be considered simultaneously [38]. For example, methods such as Combined Weighted TOPSIS [39], PT-IVHFS-TOPSIS [40], PT-VIKOR [41], and SVN-PT-TOPSIS [42] have solved multi-criteria decision making problems in several domains. In prefabrication and renovation decision-making, the evaluation criteria are often multidimensional. These include cost, construction cycle, environmental friendliness, material sustainability, and quality control. In this context, the Multi-Criteria Decision Analysis (MCDA) method provides important support to solving problems. In recent years, MCDA methods have been widely applied in the construction industry, not only in prefabricated renovation but also in other areas related to prefabricated buildings. Sánchez-Garrido evaluated the life cycle of multiple design combinations in terms of sustainability. Five well-known MCDA techniques – SAW, COPRAS, TOPSIS, VIKOR, and MIVES – were used to calculate a sustainability score for each design's life cycle performance. The study identified the design solution that achieved the best sustainability outcome [43]. Shahpari used the TOPSIS method to analyze multi-criteria decision making for productivity in residential building construction systems. The study found that management and planning criteria had the greatest impact on productivity, and cost was the most sensitive factor [44]. Hassan et al. adopted a new aggregation and ranking method that integrates the WASPAS and TOPSIS techniques. They used an approach based on semi-quadratic theory to combine rankings from different decision-making methods. The study found that cost, coordination, and standards are the most prominent barriers to the development of modular buildings [45]. Chang et al. refined the use of Pythagorean fuzzy numbers by integrating Prospect Theory with the TOPSIS method. This approach improved group decision-making in assessing risks associated with prefabricated building projects. The study found that personnel and mechanical factors are the most critical risk factors [46]. Assaf et al. developed a multi-criteria decision making (MCDM) model using Analytic Network Process (ANP) and Evidential Reasoning (ER) to select an appropriate procurement method for the off-site construction of prefabricated buildings. Their results showed that project quality, cost control, and financial arrangement were the prominent selection factors [47]. Ighravwe et al. aimed to select the most appropriate maintenance strategy for public buildings. They applied Weighted Addition and Product Assessment (WASPAS), Fuzzy Axiomatic Design Principles, Additive Ratio Assessment (ARAS), and sustainability criteria in their evaluation [48]. Yuan et al. combined Elimination, Combination, and Simplification (ECRS) techniques with intelligent simulation to develop a simulation-based optimization method for prefabricated building construction. This method considered multiple objectives and various uncertainties, reducing construction time by 5.73% and improving construction quality by 10.10% [49].

### 2.4. Application of system dynamics in the promotion of prefabricated renovation

In recent years, prefabricated decoration has attracted increasing global attention as an efficient and environmentally friendly construction technology. To promote the widespread application of prefabricated decoration, it is essential to consider influencing factors from multiple dimensions, including policy, market, technology, and management. An analytical framework is needed to reveal the relationships among these factors. System Dynamics (SD), a method for analyzing the evolution of complex systems, has been widely applied in the construction industry for policy simulation and market research [50]. System dynamics simulates the interactions among influencing factors through causal and feedback mechanisms. It helps predict the long-term effects of policy interventions or market changes on the promotion of prefabricated decoration. For example, previous studies have used system dynamics to analyze how policy incentives influence the development of prefabricated buildings. They proved that there was a positive feedback effect between the government incentive rate and the proportion of contractors adopting prefabricated buildings [51]. Li et al. combined system dynamics with evolutionary game theory to examine how policy subsidies affect the adoption of the prefabricated mode by Chinese construction enterprises [52]. Chen et al. proposed a combined modeling approach using system dynamics and agent-based modeling. They found that prefabrication technology (PT) has significant potential to reduce CO_2_ emissions during the material and construction stages of the building life cycle [53]. This result is of great significance for popularizing prefabricated decoration. Li et al. combined the methods of system dynamics (SD) and discrete event simulation (DES) to analyze the risk factors in prefabricated housing production (PHP) in Hong Kong. They found that off-site prefabrication is the most critical link [54], revealing the importance of developers in the promotion of prefabricated decoration. System dynamics provides an effective method for studying the popularization of prefabricated decoration and formulating practical, scientific guidance.

### 2.5. Literature review

Many countries have implemented a series of strategic measures aimed at promoting the development of their prefabricated and renovation industries. However, compared with developed countries such as the USA and Japan, where prefabricated decoration technology and applications are relatively mature, China still lags behind. Looking at the whole industry chain, its development is still incomplete, and there are many links to be optimized. Against the background of the Chinese government's all-out efforts to develop prefabricated decoration, many scholars have explored the multiple factors affecting the development of prefabricated decoration. However, current research generally lacks an in-depth focus on the promotion of prefabricated decoration and systematic thinking. In addition, although the development of prefabricated decoration is influenced by many complex factors, existing studies have not fully clarified the intricate relationships among them, and research based on MCDA remains limited. The specific impact of different combinations of factors on the development of prefabricated decoration, as well as the variations in their effects, has not been fully explored. Some studies have indirectly discussed the potential application of system dynamics in promoting prefabricated decoration. However, there is not enough direct research on the effective use of this method. This limits the promotion of prefabricated decoration. Therefore, a co-occurrence network can help analyze the relationships among influencing factors. This provides important support for applying the system dynamics method. System dynamics is a method that helps analyze complex factors and their interactions during the promotion process. It can be used to systematically explore strategies for promoting prefabricated decoration and provide strong theoretical support and practical guidance for its widespread adoption.

## 3. Materials and methods

### 3.1. Research framework

**Fig 1:**
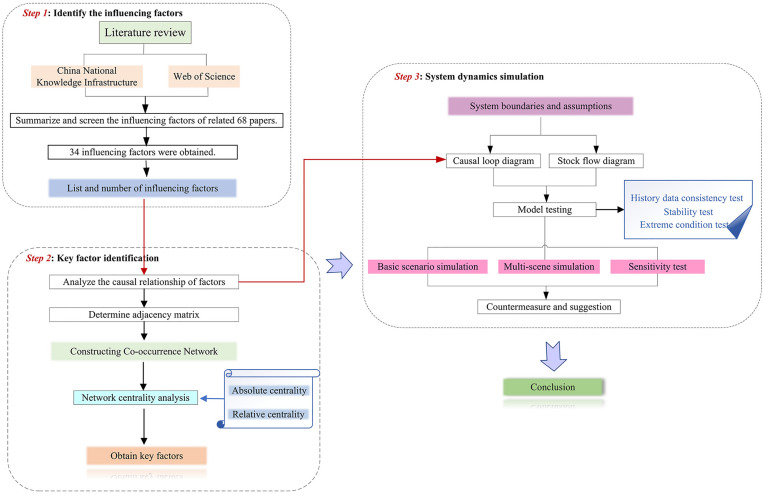
Technology road map.

This study combines co-occurrence network analysis and system dynamics to identify key factors influencing the popularization of prefabricated decoration and their causal relationships. The research framework was as shown in Fig 1.

First, based on 68 articles published in the CNKI and Web of Science databases from 2015 to 2025 on the topic of the “system dynamics”, “promotion” and “prefabricated decoration,” the influencing factors were statistically analyzed. Thirty-four factors affecting the promotion of prefabricated decoration were identified and coded through generalization and screening.

Secondly, the co-occurrence frequency of each factor in the literature was counted. An adjacency matrix was built, and a co-occurrence network diagram for the promotion of prefabricated decoration was created using Gephi software. By calculating the centrality of each factor in the network, the key factors affecting promotion were further identified.

Finally, a causality diagram and inventory flow diagram were constructed using Vensim software. After verifying the validity of the model, a basic scenario simulation, multi-scenario simulation analysis, and sensitivity test were conducted for the four key factors identified. Based on the simulation results, corresponding countermeasures and recommendations were proposed to promote the promotion and application of prefabricated decoration.

### 3.2. Systematic analysis of incentive policy for prefabricated decoration

#### 3.2.1. Policy summary.

Existing studies have shown that there is huge potential to promote prefabricated develop through policy channels in China [55]. Some prefabricated decoration policies issued by the Ministry of Housing and Urban-Rural Development of the People's Republic of China and the People's Government of Hebei Province are shown in Table 1. Based on documents from the Ministry of Housing and Urban-Rural Development, Hebei Province has issued policies suited to local conditions. These policies have further promoted the development process of prefabricated decoration in Hebei Province.

**Table 1:** Summary of policies related to prefabricated decoration and renovations.

Policy documents and standards	Concrete content	Policy support and incentives	Concrete content
Technical Standard for Interior Decoration of Prefabricated Dwellings (2021)	Specification of technical requirements for the interior decoration of prefabricated dwellings.	Priority land protection	Priority will be given to securing land for prefabricated-type building industry bases and commercial housing projects constructed in a prefabricated-type manner.
Evaluation Criteria for Prefabricated Buildings (2018)	Providing uniform standards and methods for the evaluation of prefabricated buildings.	Policy guidance	Provide incentives at 3 per cent of the above ground floor area of commercial buildings that are constructed in a prefabricated manner and have a prefabricated rate of 50 per cent or more.
Development Plan for Prefabricated Buildings in Hebei Province in the Thirteenth Five-Year Plan (2017)	By 2020, the proportion of prefabricated buildings in a new construction area is to surpass 20 percent.	Financial subsidy	Subsidies of RMB 50–100 per square meter for new prefabricated housing projects.
Implementation Opinions of the General Office of the People's Government of Hebei Province on Vigorously Developing Prefabricated Buildings (2023)	Promote collaborative construction of prefabricated building decoration and furnishings with the main structure and mechanical and electrical equipment, and promote standardized, integrated, and modular furnishing models.	Incentives	Provide incentives at 3 per cent of the above ground floor area of commercial buildings that are constructed in a prefabricated manner and have a prefabricated rate of 50 per cent or more.

#### 3.2.2. Policy analysis.

(1) Implementation status.

By 2024, the new prefabricated building area in Hebei Province reached 25.57 million square meters, accounting for 37.5% of the total new building area. This marked a year-on-year increase of 2.3 percentage points. This shows that prefabricated buildings have achieved a certain popularity, and the promotion speed of the whole house prefabricated decoration is also accelerating. In addition, seven categories of engineering cases–such as prefabricated-type decoration technology and factory production and processing of prefabricated components-were selected for the Application Case of Prefabricated-type Building Technology in Hebei Province (first batch). These cases provide reference for advanced and applicable technologies in various regions, helping to improve the application level of prefabricated building technologies.

(2) Analysis of challenges and problems. Although Hebei Province has introduced several policies to promote prefabricated decoration, the current incentive measures remain relatively limited, particularly in tax benefits and the streamlining of the approval process. The policy limitations may affect the further development of the prefabricated decoration industry. Therefore, it is recommended to build on existing policies by increasing tax incentives, streamlining the approval process, and offering more diversified incentives to encourage enterprises to adopt prefabricated decoration. In addition, current financial support is limited. It is recommended to set up a special fund to support R&D and innovation in prefabricated decoration technologies, enhance the core competitiveness of local enterprises, and accelerate industry development.

### 3.3. Identification of influencing factors

Systematic review of existing literature to identify relevant factors is an effective method commonly used in academic research. The effective promotion of prefabricated decoration requires systematic identification and integration of its influencing factors. This helps clarify key constraints and improve both the efficiency and practical feasibility of promotion. Therefore, this study conducted a literature search and screening in the CNKI and Web of Science databases for the period 2015–2025, using the keywords “prefabricated decoration”, “promotion”, and “system dynamics”, in order to provide a solid theoretical foundation for constructing the influencing factor system. Firstly, 82 documents were obtained by keyword search. Subsequently, a preliminary screening was conducted based on titles and abstracts to ensure strong relevance to the research topic. After literature that did not consider key stakeholders or their influence mechanisms was excluded, a total of 68 articles were finally selected as the research basis for analyzing the influencing factors of prefabricated decoration promotion. In the process of identification and selection, the potential influencing factors mentioned in the literature were extracted by structured content analysis. To ensure the significance and reliability of the selected factors, a double inclusion criterion was applied. A factor was included if it appeared in at least two independent documents with confirmation. A factor was also included if it appeared in only one document but was supported by solid quantitative or qualitative evidence. Finally, 34 influencing factors were identified and classified according to the stakeholder framework of Han et al. [56] and the initial system of influencing factors for prefabricated decoration promotion was formed, as shown in Table 2.

**Table 2:** Influencing factors in the initial system.

Stakeholders	Influential factors	Source
Government	G1 Government support	[57,58]
	G2 Government propaganda	[59]
	G3 Government supervision	[60,61]
	G4 Incentive policy	[62]
	G5 Financial subsidy	[63,64]
	G6 Hebei Province GDP total amount	[65]
	G7 Hebei Province's total population	
	G8 Per capita GDP	
	G9 Per capita disposable income	
Industry associations	I1 Industry standards	[66]
	I2 Industry development plan	
Supervision agency	S1 Decoration price.	[67]
	S2 Decoration price income ratio.	
Developers	D1 Technical level	[68–71]
	D2 Standardization of components	[72]
	D3 Degree of improvement in technical standards	[73]
	D4 Scope of BIM technology	[74]
	D5 Professional and technical talent level	[75]
	D6 Total market supply	[76]
	D7 New area of prefabricated decoration	[77]
	D8 Decoration efficiency	[78]
	D9 Integrity of the industrial chain	[79]
	D10 Enterprise management model	[80]
	D11 Enterprise decoration experience	
	D12 Use of recycled packaging materials	[81]
	D13 The amount of waste generated	[82,83]
	D14 Participants in collaboration	[84]
	D15 Developers’ willingness to use	[71,85]
Consumers	C1 Consumers’ willingness to use	[86–88]
	C2 Sustainable development appeal	[89]
	C3 Total market demand	[90]
Material Suppliers	M1 Material quality and acceptance	[91]
	M2 Standardization of universal interfaces	
	M3 Equipment pipeline is easy to disassemble	

### 3.4. Co-occurrence network analysis of influencing factors

#### 3.4.1. Co-occurrence network construction.

Co-occurrence analysis is a method to quantitatively analyze the potential relationship between two elements by calculating the frequency of the two elements appearing together. The co-occurrence network method has been applied in many fields, such as finance and supply chain. However, it has few applications in prefabricated decoration, and there is little literature using centrality classification to determine key factors. In order to study the relationship between the factors influencing prefabricated decoration promotion, we used Gephi software to analyze its co-occurrence network. When two groups of influencing factors appear at the same time, it is recorded as 1; when they do not appear at the same time, it is recorded as 0. After analyzing 68 related studies, the adjacency matrix of influencing factors of prefabricated decoration was obtained as shown in Table 3.

**Table 3:** Adjacency matrix.

	G1	G2	G3	...	M1	M2	M3
G1	0	15	6	...	12	0	7
G2	15	0	5		1	0	1
...	...	...	...	...	...	...	...
M3	7	1	0	...	0	0	0

*The complete adjacency matrix is placed in the supporting information.

**Fig 2:**
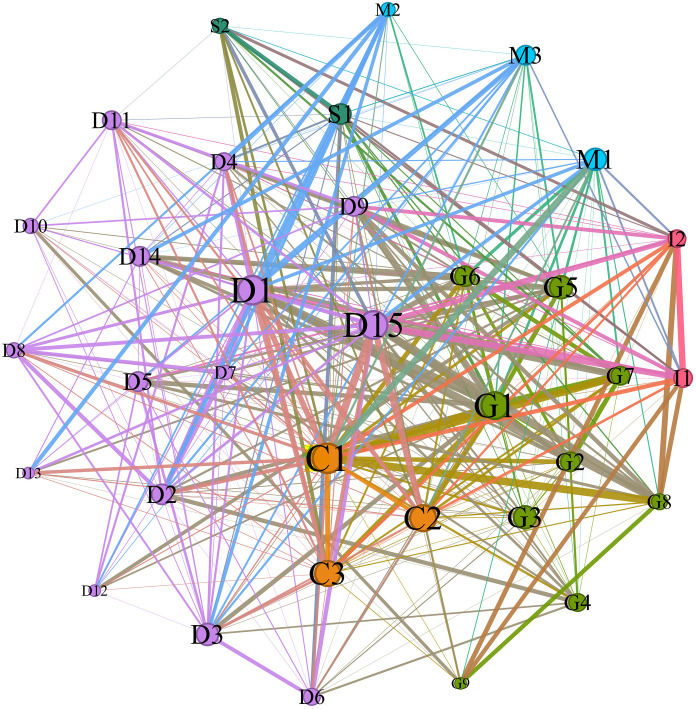
Co-occurrence network of influencing factors of prefabricated decoration.

Based on the adjacency matrix, we used Gephi software to draw a co-occurrence network diagram of the influencing factors of the prefabricated decoration. The network is an undirected weighted network (see Fig 2). Differently colored circles represent various categories of influencing factors. The connecting lines indicate relationships between the factors, and the thickness of the lines reflects the frequency of co-occurrence. The darker the color, the greater the weight. The size of the circle represents the importance of the influencing factor and reflects the strength of its connection to other factors. For example, C1 (consumers’ willingness to use) has the largest circle, indicating its high importance in the network and its close relationship with other factors.

#### 3.4.2. Centrality analysis of factors influencing prefabricated decoration.

Of the two equations below, the former represents the total number of direct contacts between node i and other n−1 nodes. The latter represents the ratio of the absolute centrality of node i to the maximum possible continuous number of other n−1 nodes, as follows:


$$C_{D}(N_{i})=\sum_{j=1}^{n}x_{ij}(i\neq j)$$ (1)

$$C\prime_{D}(N_{i})=\frac{C_{D}(N_{i})}{n-1}$$ (2)

$C_{D}(N_{i})$ in Equation (1) represents the absolute centrality of node i and $\sum_{j=1}^{n}x_{ij}$ represents the number of direct connections between i node and other j nodes. Equation (2) indicates the relative centrality of nodes. $CD(N_{i})\prime$ in Equation (2) represents the relative centrality of i to the node.

According to the research of [92–94], the first three to five risk factors measured in social network analysis (SNA) are often selected as important risk factors. Complex networks and social networks have similar network characteristics. Therefore, based on this theoretical framework, this paper makes a comprehensive analysis of the influencing factors in the process of prefabricated decoration promotion, and determines the key factors. First of all, government support (G1) has been identified as a key factor in the study of prefabricated decoration. Because government policies can effectively reduce market access barriers, promote the improvement of technological level, and promote the wide application of prefabricated decoration through policy incentives. Secondly, the improvement of technical level (D1) is an important factor affecting the promotion speed and market acceptance of prefabricated decoration. It helps ensure decoration quality and construction efficiency. It also drives market recognition through innovation, which speeds up its adoption. In addition, the developer's willingness to use (D15) plays a key role in promoting prefabricated decoration in the market. The developers’ adoption intention directly affects the wide application of this decoration method, especially on the basis of cost-effectiveness and long-term development potential. Developer support can greatly promote market adoption rates. At the same time, consumers’ willingness to use (C1), as an important indicator to measure market acceptance, determines whether prefabricated decoration can be recognized by the final market. Changes in consumer demand directly affect the popularity of prefabricated decoration. Therefore, consumers’ willingness to use is regarded as one of the core factors. Although the sustainable development appeal (C2) occupies an important position in the process of prefabricated decoration promotion, as a macro-development goal, it is more realized through technological progress and policy support. Indeed, government support (G1) already encompasses sustainable development aspirations in its policy framework, so there is no need to single them out as a key factor. Based on the above analysis, this paper chooses C1, D1, D15 and G1 as the key factors in the research, aiming to systematically reveal the key driving factors affecting the promotion of prefabricated decoration. The other factors are divided into general factors. The remaining factors are divided into general factors, and the specific results are shown in Table 4.

**Table 4:** Centrality grading results of influencing factors of prefabricated decoration promotion.

Rank	Factor	Absolute centrality	Relative centrality	Centrality ratio	Grade
1	C1	32	0.970	0.047	Key factor
2	D1	31	0.939	0.045	
3	D15	30	0.909	0.044	
4	G1	29	0.879	0.042	
5	C2	28	0.848	0.041	General factors
6	C3	27	0.818	0.039	
7	G3	24	0.727	0.035	
8	G5	24	0.727	0.035	
9	C3	23	0.697	0.034	
10	M1	23	0.697	0.034	
11	G6	21	0.636	0.031	
12	S1	21	0.636	0.032	
13	D2	21	0.636	0.034	
14	G2	20	0.606	0.029	
15	D5	20	0.606	0.029	
16	D9	20	0.606	0.029	
17	D14	20	0.606	0.029	
18	M3	20	0.606	0.029	
19	G7	19	0.576	0.028	
20	I1	19	0.576	0.028	
21	D11	19	0.576	0.028	
22	G4	18	0.545	0.026	
23	I2	18	0.545	0.026	
24	D4	18	0.545	0.026	
25	D6	17	0.515	0.025	
26	G8	16	0.485	0.023	
27	S2	15	0.455	0.022	
28	D8	15	0.455	0.022	
29	D10	15	0.455	0.022	
30	D7	14	0.424	0.020	
31	M2	14	0.424	0.020	
32	G9	11	0.333	0.016	
33	D12	11	0.333	0.016	
34	D13	11	0.333	0.016	

### 3.5. System dynamics

System dynamics, first proposed by Jay W. Forrester in 1961, is an analytical tool for studying the interactions among variables and for exploring solutions to complex nonlinear systems [95,96]. By analyzing the interrelationships among multiple factors, system dynamics reveals feedback mechanisms and causal structures. This approach offers a new analytical perspective and provides data support for optimizing promotion strategies. Although widely applied in many fields, its use in the study of prefabricated decoration remains limited. Taking Hebei Province as a case study, this paper applies system dynamics to the complex system of prefabricated decoration promotion, addressing current challenges and providing a decision-making basis for relevant stakeholders.

#### 3.5.1. System boundaries and assumptions. (1) Time boundary.

In 2016, the “Guiding Opinions of the General Office of the State Council on Vigorously Developing Prefabricated Buildings [2016] No. 71” emphasized the need to enhance prefabricated decoration and promote menu‑style full decoration to meet individual consumer needs. The most recent policy, the Peak Carbon Dioxide Emissions Implementation Plan for Urban and Rural Construction in Hebei Province, sets the target that by 2030 prefabricated buildings will account for 40% of new urban buildings. Therefore, this study defines the time boundary as 2016–2030 with a one‑year time step.

(2) Spatial boundary

The 13th Five-Year Plan of Prefabricated Buildings in Hebei Province states that, by 2025, prefabricated buildings will account for more than 30% of new buildings. The prefabricated construction method will become one of the main construction methods. As an important part of the coordinated development of Beijing-Tianjin-Hebei, the application scope of prefabricated decoration in Hebei Province still needs to be improved. Therefore, taking Hebei Province as an example, this paper discusses the promotion of prefabricated decoration in order to provide a reference for relevant policy formulation and industry development.

#### 3.5.2. Causality diagram.

**Fig 3:**
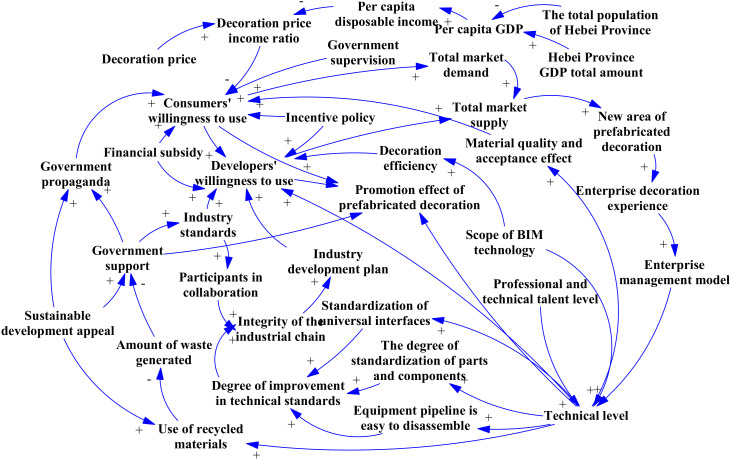
Causal loop diagram.

A causality diagram is often used to explain the relationships between elements and their dynamic feedback processes. It helps conceptually understand a system and provides the basis for creating a stock flowchart [97]. In this diagram, each node represents a variable, connected by directed causal arrows [98]. When a causal chain loops back to its originating variable, it forms a feedback loop [99], which can be reinforcing (“+”) or balancing (“–”) depending on the direction of influence [100]. By analyzing the interactions between the influencing factors of prefabricated decoration promotion, Vensim drew the causal loop diagram shown in **Fig 3**. There are four key loops in the causality diagram, which are respectively three positive feedback loops and one negative feedback loops.

(1) Positive feedback loop

Developers’ willingness to use → (+) total market supply → (+) new area for prefabricated decoration → (+) enterprise decoration experience → (+) enterprise management mode → (+) technical level → (+) standardization degree of parts and components → (+) perfection degree of technical standards → (+) perfection degree of industrial chain → (+) industry development plan → (+) developers’ willingness to use.

The developers’ willingness to use is the core factor in promoting the development of the prefabricated decoration market. As developers’ willingness to use increases, market supply will grow. This will promote the expansion of the new area of prefabricated decoration. This growth further provides decoration experience for enterprises, thus optimizing the management mode of enterprises and improving the technical level. These technological advances have promoted the standardization of parts and components and improved technical standards. At the same time, they have also promoted the optimization and development of the industrial chain. The further development of the industrial chain provides support for the implementation of industry planning. Finally, this series of changes enhances the developer's willingness to use and forms a positive feedback loop, thus accelerating the popularization and application of prefabricated decoration.

Technical level → (+) standardization degree of parts and components → (+) perfection degree of technical standards → (+) perfection degree of industrial chain → (+) industry development planning → (+) developers’ willingness to use → (+) total market supply → (+) newly added area of prefabricated decoration → (+) enterprise decoration experience → (+) enterprise management mode → (+) technical level.

The improvement of the technical level is one of the key factors in the development of prefabricated decoration. As the technical level improves, the standardization and technical standards of parts and components also improve. This promotes the optimization and development of the industrial chain. These improvements support the implementation of the industry development plan and further enhances the developer’s willingness to use it. An increase in developers’ willingness to use has led to greater market supply. This, in turn, has promoted the expansion of the new area of prefabricated decoration. The growth of the new area has led to more decoration experience for enterprises and promoted the optimization of the enterprise management mode. Finally, this series of improvements further enhanced the technical level, accelerating the popularization and development of prefabricated decoration.

Consumers’ willingness to use → (+) total market demand → (+) total market supply → (+) new area of prefabricated decoration → (+) enterprise decoration experience → (+) enterprise management mode → (+) technical level → (+) material quality and acceptance effect → (+) consumers’ willingness to use.

Consumers’ willingness to use is important for promoting the development of the prefabricated decoration market. When consumers’ willingness to use increases, total market demand rises. This drives an increase in market supply and further promotes the expansion of the new area of prefabricated decoration. With the expansion of the new area, enterprises accumulate more experience with the decoration process, thus optimizing the management mode and improving the technical level. Improving the technical level enhances material quality and acceptance. This increases consumer trust, creates a positive feedback loop, and strengthens their willingness to use prefabricated decoration. In the end, the promotion of consumers’ willingness to use encourages the growth of market demand and supply, and promotes the wide application of prefabricated decoration.

(2) Negative feedback loop

Government support → (+) government publicity → (+) consumers’ willingness to use → (+) developers’ willingness to use → (+) total market supply → (+) new area of prefabricated decoration → (+) enterprise decoration experience → (+) enterprise management mode → (+) technical level → (+) scope of use of recyclable packaging materials → (-) amount of waste → (+) government support.

Government support has increased consumers’ willingness to use by strengthening publicity. This, in turn, has raised developers’ willingness to adopt prefabricated decoration. The developers’ willingness to use has promoted an increase in the total market supply and the expansion of the new area of prefabricated decoration. The growth of the newly added area has enabled enterprises to accumulate more experience in the decoration process, thus optimizing the management mode and technical level of the enterprise. The improvement of technical level promotes the use of recyclable packaging materials. This helps reduce the amount of waste. The reduction in waste, in turn, further attracts government support and forms a negative feedback mechanism. Nevertheless, reducing waste and achieving sustainable development goals will guide the prefabricated decoration industry toward a more environmentally friendly and efficient path.

#### 3.5.3. Stock flowchart.

**Fig 4:**
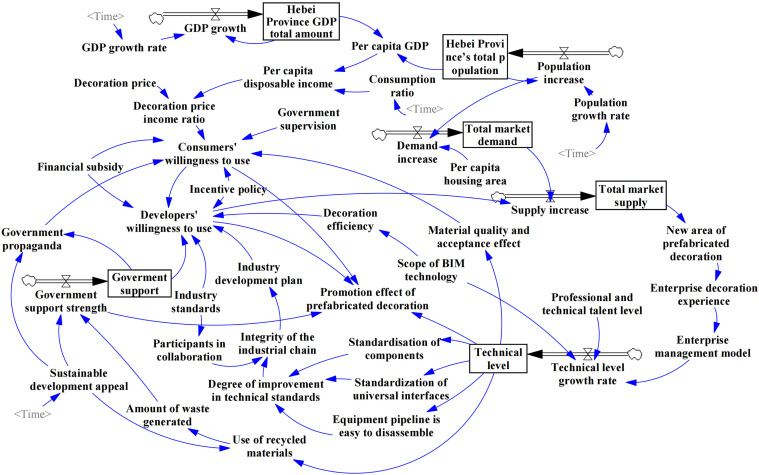
Stock flow diagram.

A stock flowchart is used to quantitatively analyze system behavior under different conditions and to clarify the structure and dynamic characteristics of the system [101]. Based on the causal loop diagram, variables are categorized into level, rate, auxiliary, and constant types [102]. Level variables represent the system's state or accumulation, rate variables describe the rate of change of level variables, auxiliary variables serve as intermediate explanatory factors, and constant variables take fixed values (see Table S3). To enhance realism, time parameters based on historical data were incorporated into GDP growth rate, consumption ratio, population growth rate, and sustainable development appeal. The causal loop diagram was simplified considering data availability and rationality, and Vensim was used to construct the stock flowchart shown in Fig 4.

#### 3.5.4. Formula construction.

The basic data in the model comes from Hebei Statistical Yearbook (Hebei Provincial Bureau of Statistics, 2023). The simulation starts and ends from 2016 to 2030, and the default step length is 1 year. The key equations are illustrated below, and all equations are shown in the supporting information S3 Table.

(1) Economic level

Gross GDP and urban population are important indicators reflecting the level of economic development in a certain region.

Total urban GDP = INTEG (GDP growth, 28474.1).

Total urban population = INTEG (population growth, 6618).

(2) Sustainable development demands

With the “double carbon” policy and the air pollution protection law and other laws and regulations put forward, people's demands for sustainable development will continue to improve. Therefore, this variable is set as a table function that changes with time, and the equation is as follows.

#### Sustainable development appeal = WITH LOOKUP (Time, ([(2016,0) – (2030,1)], (2016,0.003), (2017,0.12), (2018,0.15), (2019,0.2), (2020,0.22), (2021,0.24), (2022,0.3), (2025,0.34), (2028,0.38), (2030, 0.4))).

(3) Per capita disposable income

**Fig 5:**
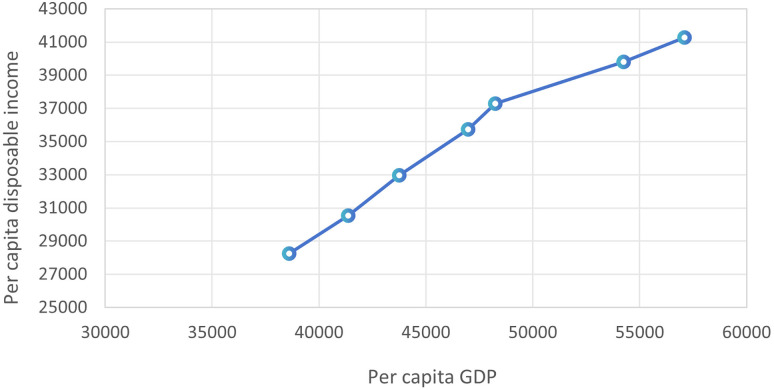
Regression analysis chart of per capita GDP and per capita disposable income.

In this study, SPSS was used for the regression analysis of per capita disposable income and per capita GDP. The results show that p = 0.00 < 0.01, indicating that they are significantly correlated at the level of 0.01, and the R-squared value is 0.968. The regression equation is as follows, and the regression line is shown in Fig 5.

#### 3.5.5. Model test.

The validity test of the model was realized by comparing the output values of the variables in the model with the actual values of the variables. There should be no significant difference between the model results and the actual results to ensure consistency between the model and the real system [103]. We tested the validity of the model through authenticity, stability, and extreme case tests.

(1) Historical data consistency test

**Fig 6:**
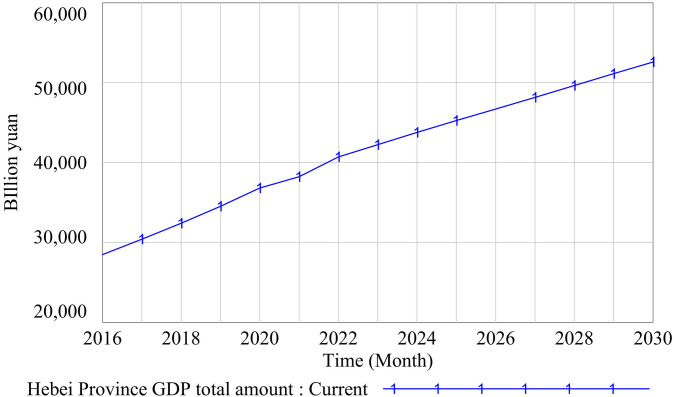
Historical data consistency test 1 results.

**Fig 7:**
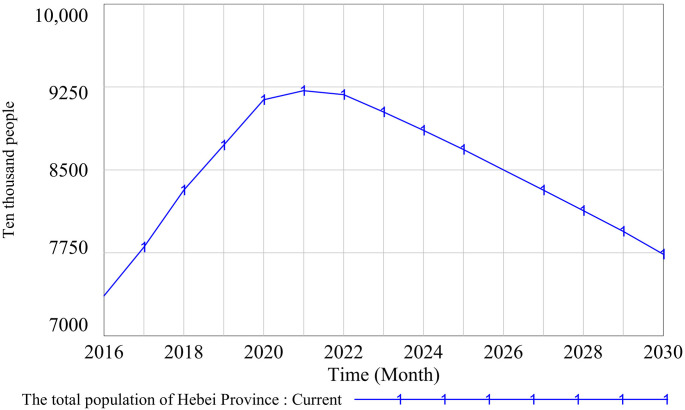
Historical data consistency test 2 results.

A consistency test on historical data checks the fitting degree between the historical values of key variables and the simulation values in a model. That is, the validity of a model is judged by comparing the differences between the simulation values of system-level variables and historical statistical data [104]. In this paper, the historical data consistency test is carried out on the two variables of the total GDP of Hebei Province and the total population of Hebei Province. The validity of the model is verified. The data source was the statistical yearbooks issued by Hebei Provincial Bureau of Statistics from 2016 to 2022. The historical data and simulation results are shown in Table 5 and Table 6, and the simulation results are shown in Fig 6 and Fig 7. Although the fitting stability of each variable was different, the total error value was less than 0.5%. This shows that the fitting degree between the running results of the model and the actual data was high. The model can accurately describe the basic state of the research system, has a good prediction effect, and can be simulated in the next stage.

**Table 5:** Consistency test of historical GDP data.

Year	2016	2017	2018	2019	2020	2021	2022
Gross GDP (billion yuan)	28474.1	30640.8	32494.6	34978.6	36013.8	40397.1	42370.4
Analog value	28452.9	30638.6	32488.6	34978.6	36028.5	40419.7	42370.4
Error(%)	−0.070	0.007	−0.002	0	0.041	0.056	0

**Table 6:** Consistency test of historical data of total population in Hebei Province.

Year	2016	2017	2018	2019	2020	2021	2022
Total population (ten thousand people)	7375.0	7409.1	7426.5	7446.6	7463.9	7448.0	7420.0
Analog value	7375.0	7421.0	7434.4	7468.8	7453.2	7447.5	7423.3
Error(%)	0	0.161	0.106	0.298	−0.143	0.007	0.045

(2) Stability test

**Fig 8:**
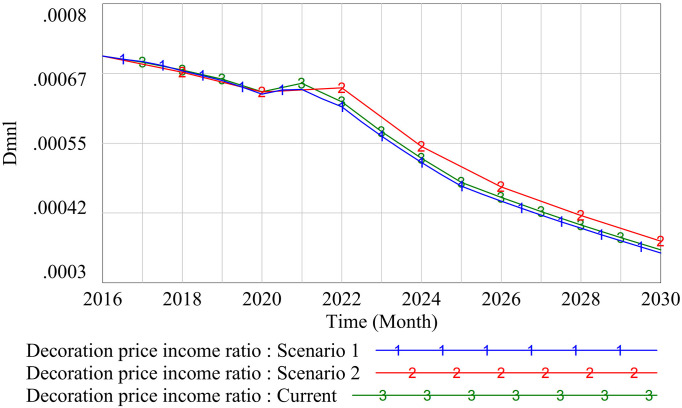
Stability test results.

A stability test tests the stability of a model by changing the time step. We selected the decoration price – income ratio variable for the simulation test, set the TIME STEP of scenarios 1 and 2 to 0.5 and 2, respectively, and tested the stability of the model compared with the original state Current with a TIME STEP of 1. The results are as shown in Fig 8. The results show that the behavior trend of the decoration price – income ratio is still consistent after changing the time step, indicating that the model is relatively stable.

(3) Extreme case test

**Fig 9:**
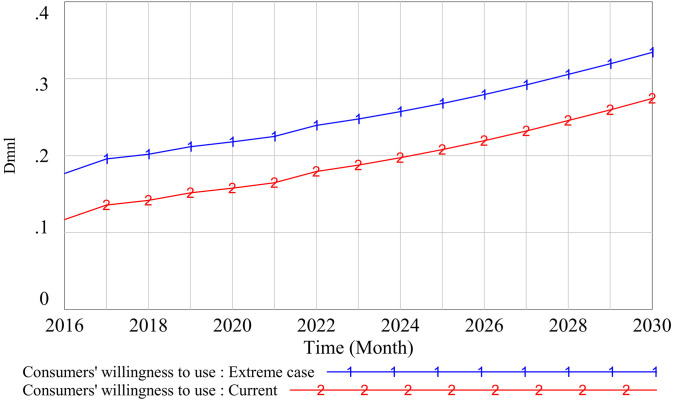
Test results of extreme cases.

The extreme case test selects a suitable constant, adjusts its value to a limit of 1, and observes whether the curve change of the simulated variable is the same as the original trend when the value is adjusted to the limit value. We adjusted the government supervision from 0.4 to the limit value of 1 and observed the changes in the consumers’ willingness to use curve. The results show that, even if government supervision is adjusted to the limit value of 1, consumers’ willingness to use is the same as the original curve. Therefore, the model passes the extreme test, the results are shown in Fig 9.

## 4. Results and discussion

The model test results show that the system has high stability and availability. In the following analysis, Vensim software is used to simulate four key influencing factors under multiple scenarios. The future development trend of prefabricated decoration in Hebei Province is examined by comparing the outcomes across these scenarios. In the figures, the values for 2022 and 2030 are highlighted to present the key data points. These values also illustrate the variable trends at the critical policy implementation period and the end of the forecast period.

### 4.1. Basic scenario simulation of the prefabricated decoration market

**Fig 10:**
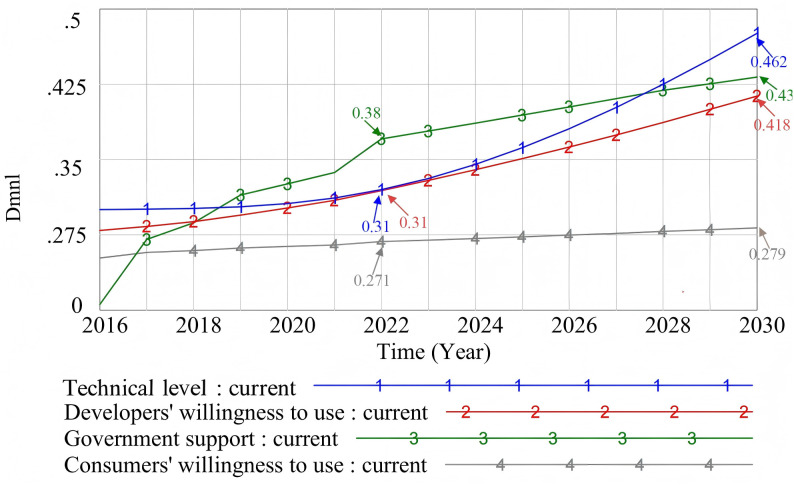
Basic scenario simulation.

Fig 10 shows the simulation and prediction results of four key factors: technical level, developers’ willingness to use, government support, and consumers’ willingness to use. As shown in Fig 10, with the promotion of relevant policies, the technical level of prefabricated decoration shows a trend of increasing first slowly and then rapidly. The willingness of developers to adopt prefabricated decoration shows a more significant growth trend. On the one hand, the government has defined the prefabricated rate requirements of new buildings through mandatory policies. On the other hand, it has also introduced a series of incentive preferential policies. These comprehensive implementation of these policies and measures has increased the willingness of developers [105]. The growth rate of government support was first fast and then slow, and tends to be stable in the middle and late stages. Especially before 2020, the popularity of prefabricated decoration in Hebei Province was low, and the government introduced a large number of policies to promote prefabricated decoration. However, with the increasing popularity of prefabricated decoration, the number of relevant policies will gradually decrease. But consumers’ willingness to use prefabricated decorations continues to grow steadily. This is because consumers consider many factors when choosing decoration methods, and these factors are less affected by mandatory policies. Therefore, although the increase in consumer willingness is relatively slow, it still shows a steady and stable growth trend.

### 4.2. Multi-scenario simulation of key factors influencing prefabricated decoration

#### 4.2.1. Government support. The demand for sustainable development (Scenario 1) and waste production.

**Fig 11:**
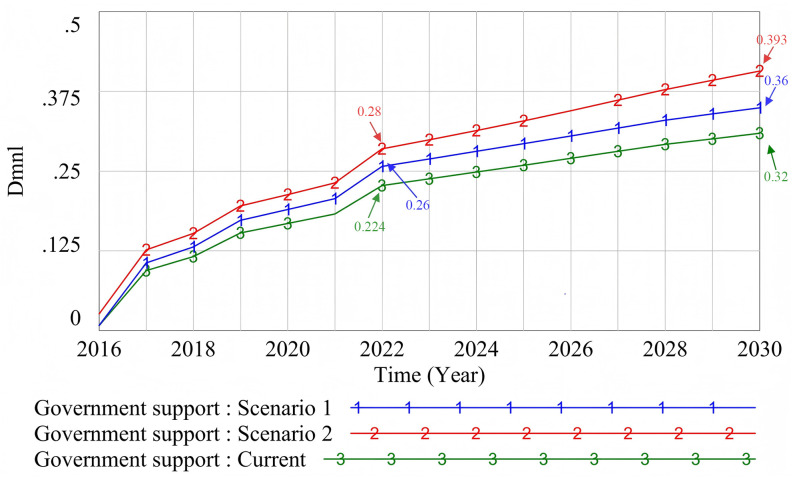
The influence of different scenarios on government support.

(Scenario 2) are simulated, and the original state is “Current.” The simulation results are shown in Fig 11.

When comparing the impact of government support on waste production (Scenario 2) with the impact of sustainable development demands (Scenario 1), the former shows a more significant effect. This phenomenon reflects the practical considerations in the process of policy formulation: the use of a prefabricated decorative floor system has obvious carbon reduction potential [106], and sustainable development is also a common pursuit of the public. However, at the government decision-making level, the reduction of waste production as a direct result of the application of prefabricated decoration, more intuitively reflects the effectiveness of the policy implementation, thus reinforcing the government's support. Poplar, as a fast-growing and renewable source of wood, can also be used as an integrated green structural decoration material to excellent effect. It contributes to environmental protection. The government should prioritize the quantitative evaluation of policy effects when formulating and optimizing policies in the future, so as to promote the wide application and sustainable development of environmental protection technologies such as prefabricated decoration.

#### 4.2.2. Developers’ willingness to use.

**Fig 12:**
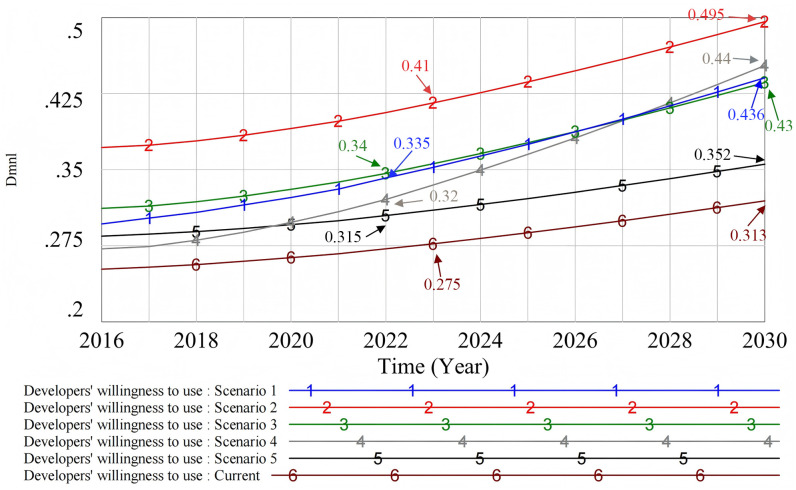
The influence of different scenarios on developers’ willingness to use.

The consumers’ willingness to use (Scenario 1), financial subsidy (Scenario 2), decoration efficiency (Scenario 3), policy support (Scenario 4), and industry development planning (Scenario 5) were simulated. The original state is “Current,” and the simulation results are shown in Fig 12.

Wu's research found that economic incentive policies and mandatory implementation policies can influence developers through perceived usefulness [107]. Our research shows that, among the many factors that affect developers’ willingness to use prefabricated decoration, financial subsidies (Scenario 2) have the most significant positive effect. In contrast, the improvement of consumers’ willingness to use (Scenario 1) and the improvement of decoration efficiency (Scenario 3) have a positive impact on developers’ willingness to use, but not as significantly. This shows that although consumers’ willingness to use has increased, it may take a long time for their rising purchasing power to significantly influence developers’ willingness to use prefabricated decoration. In addition, although higher decoration efficiency helps shorten the construction period, it is not the most effective strategy for increasing willingness. This is because it has limited impact on reducing costs for developers. Additionally, the industry development plan (Scenario 5) is less attractive to developers. Because the industry development plan targets overall development at the macro level, its impact on developers is indirect. Therefore, it is less attractive than measures that directly reduce costs or increase returns. Moreover, its contribution to the promotion of prefabricated decoration is limited, indicating that the current industry development plan is still insufficient to encourage developers to adopt the new technologies. It is worth noting that although policy support (Scenario 4) has less direct impact on developers’ willingness to use in the early stage of promotion, its long-term effect emerges gradually and has a strong, lasting influence on their willingness to use prefabricated decoration. This discovery emphasizes the key role of continuity and stability of policy support in promoting technological innovation in the industry. It also suggests that policy makers should focus on long-term planning and phased implementation of policies in order to maximize the policy effect.

#### 4.2.3. Technical level.

**Fig 13:**
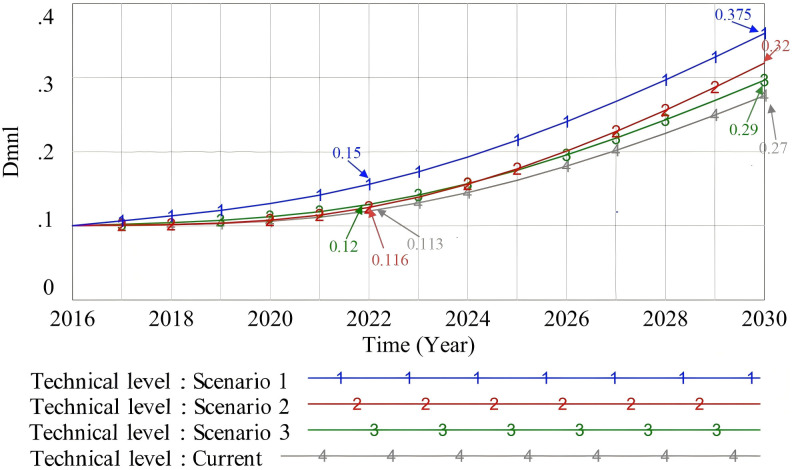
The influence of different scenario changes on the improvement of the technical level.

The application scope of BIM technology (Scenario 1), the enterprise management mode (Scenario 2), and the professional and technical personnel level (Scenario 3) were simulated. The original state is “Current,” and the simulation results are shown in Fig 13.

The application scope of BIM technology (Scenario 1) shows the most significant positive effect on technology promotion. By consulting a comprehensive information model, developers can find and solve problems in the early stage of prefabricated decoration. This helps reduce the sunk costs caused by design or construction errors and ensures sustainable buildings [108]. This discovery shows that BIM technology plays an important role in improving decision-making quality and risk management in the early stage of a project. In the late stage of prefabricated decoration promotion, Shang’s research shows that the proportion of technical personnel has the greatest impact on industrial efficiency at the micro level. However, we found that optimizing the management mode at the enterprise level (Scenario 2) improves the operational efficiency of the project team more effectively than simply improving the level of professional and technical personnel (Scenario 3). Optimizing the management mode promotes efficient resource allocation and team coordination. It also improves communication and cooperation, providing strong support for ongoing technical improvement.

#### 4.2.4. Consumers’ willingness to use.

**Fig 14:**
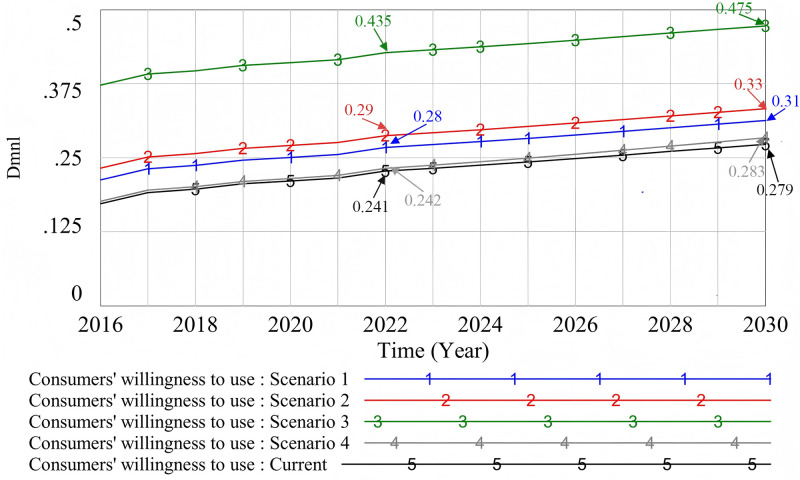
The influence of different scenarios on consumers’ willingness to use prefabricated decoration.

The material quality and acceptance effect (Scenario 1), incentive policies (Scenario 2), financial subsidies (Scenario 3), and government supervision (Scenario 4) were simulated. The original state is “Current,” and the simulation results are shown in Fig 14.

As the most direct economic incentive measure, financial subsidies (Scenario 3) have a significant incentive effect on consumers. This discovery highlights the key role of economic incentives in guiding consumer behavior, especially in the initial stage of promoting the popularization of emerging decoration methods. Therefore, in order to promote the promotion of new decoration methods, the most effective measure is still to provide targeted financial subsidies. Through financial subsidies, consumers are significantly more likely to choose prefabricated decoration. This shows that consumers will weigh the economic costs and personal interests in the decision-making process. Even if financial subsidies decrease or change, consumers may still prefer prefabricated decoration after experiencing its advantages. This reflects the dynamic nature of consumer preferences. It can be seen that the role of subsidies is not limited to short-term economic incentives, but may also affect consumers’ long-term preferences through direct experience. Pan et al.’s research pointed out that the government's subsidies to consumers have a certain “threshold effect”. After exceeding this threshold, further subsidies will not significantly promote the development of prefabricated buildings [109]. However, the research in this paper shows that government incentive policies (Scenario 2) have a sustained and significant impact on improving consumers’ willingness to use in a long time. Policies like tax and loan incentives have increased market acceptance of prefabricated decoration. They do so by reducing consumers’ economic burden or boosting their sense of benefit. This phenomenon shows that consumer decision-making is influenced by economic factors and external incentives. It also highlights the regulatory role of policies in shaping consumer behavior and preferences. In addition, material quality and acceptance effect (Scenario 1), as one of the core elements influencing consumers, directly affects consumers’ decisions. Traditional decoration has many problems, especially potential health risks for residents. These issues increase the likelihood that people will choose prefabricated decoration. High-quality materials and strict standards can significantly enhance consumers’ trust and satisfaction [110][110], thus enhancing their willingness to use prefabricated decoration. This phenomenon shows that consumers value not only economic benefits but also actual performance and long-term advantages when making decisions. It is worth noting that although the impact of government supervision (Scenario 4) is relatively limited, building a supervision system and improving the reward and punishment mechanism can increase public trust. This indirectly enhances consumers’ willingness to use. Consumers’ trust in the government plays a vital role in their acceptance of policies and confidence in products. Therefore, government regulation indirectly promotes the market penetration of prefabricated decoration by strengthening consumers’ trust. In summary, when consumers choose prefabricated decoration, they pay more attention to economic incentives, policy preferences, and product quality and effectiveness than government supervision. These factors are closely related to consumers’ direct experience and expectations of long-term benefits. Therefore, an in-depth understanding of consumers’ behavioral preferences – especially the decision-making process in the face of different incentives and product quality – is crucial for promoting the widespread application and marketization of prefabricated decoration.

#### 4.2.5. Simulation analysis of the popularization effect of key influencing factors.

**Fig 15:**
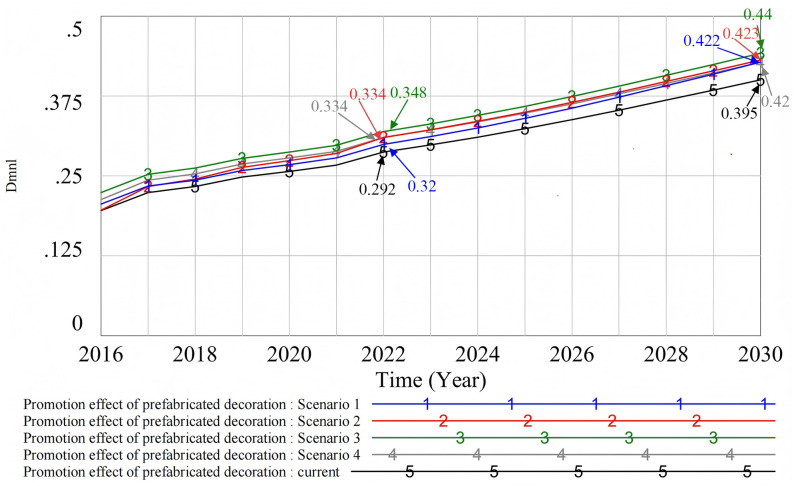
Simulation analysis of the popularization effect of key influencing factors.

After the four key variables were upgraded to the same proportion, their influence on the promotion of prefabricated decoration was observed (see Fig 15). Among them, the increase in the developer’s willingness to use (Scenario 3) has the most significant impact on the promotion of prefabricated decoration. This is because developers are the main executors of policies. Their promotion behavior is influenced not only by policy requirements but also by factors like material prices and government subsidies. Therefore, their willingness plays a key role in the effectiveness of promotion. In contrast, improving consumers’ willingness to use (Scenario 4) and government support (Scenario 2) have similar effects. They can promote the popularization of prefabricated decoration, but the impact is limited. The role of upgrading the technical level (Scenario 1) is relatively limited, mainly because the direct effect of technical upgrading on consumers is weak. At the same time, this may increase costs. As a result, it can weaken developers’ enthusiasm and have a limited impact on the promotion of prefabricated decoration.

#### 4.2.6. Comparison of the development trend of key influencing factors under different policies.

**Fig 16:**
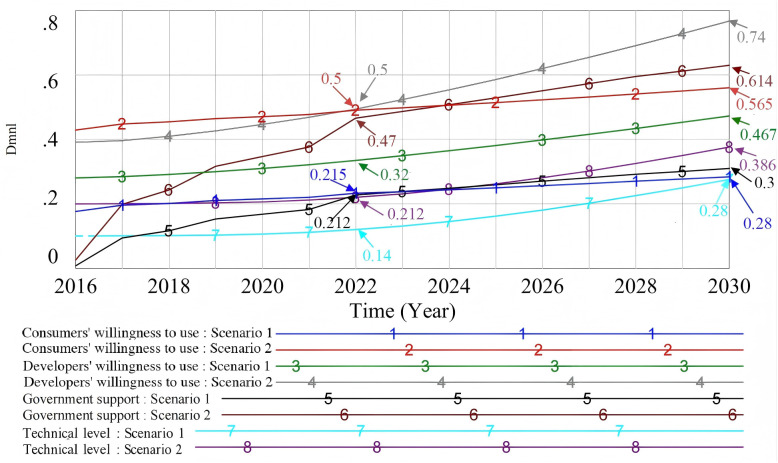
Comparison of the development trends of key influencing factors under different policies.

In the promotion process of prefabricated decoration, government policy is still the main driving force. In order to compare the development trend of key influencing factors under different policies, this paper sets two scenarios: scenario 1 is weak incentive policy, and scenario 2 is strong incentive policy. According to the analysis of Fig 16, the weak incentive policy leads to limited government support, slow technological progress, and a low technical level. As a result, developers and consumers lack acceptance and confidence in prefabricated decoration. This reduces the effectiveness of promotion. In contrast, under the strong incentive policy, government support for technology research and development has increased. The technical level has improved significantly. Developers and consumers show stronger recognition and willingness to use prefabricated decoration. In particular, developers’ willingness to participate has greatly increased. As a result, the promotion effect of prefabricated decoration has improved significantly.

### 4.3. Sensitivity analysis

In order to evaluate the sensitivity of the promotion of prefabricated decoration to economic fluctuations and policy changes, we carried out a univariate sensitivity analysis and a multivariate sensitivity analysis. These analyses aim to quantify the specific roles of economic fluctuations and policy changes.

#### 4.3.1. Univariate sensitivity test. (1) Sensitivity test of prefabricated decoration cost.

**Fig 17:**
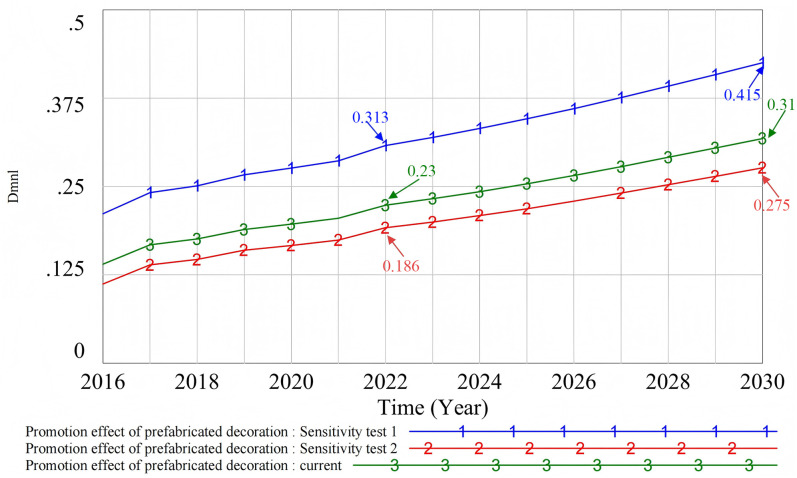
Sensitivity test of prefabricated decoration costs.

The price test of prefabricated decoration (Fig 17) shows that when the price drops (Sensitivity test 1), developers’ income increases. This greatly boosts their willingness to use, promotes the spread of prefabricated decoration, and significantly enhances the promotion effect. In this case, the promotion effect is highly sensitive to cost changes. Relatively speaking, when the decoration price rises (Sensitivity test 2), developers’ income decreases. However, due to mandatory policy requirements and consumers’ continued demand for livable environments, although the promotion effect has been delayed to a certain extent, the degree of change has been relatively limited, and the overall promotion effect has remained at a relatively stable level.

(2) Sensitivity test of the number of incentive policies

**Fig 18:**
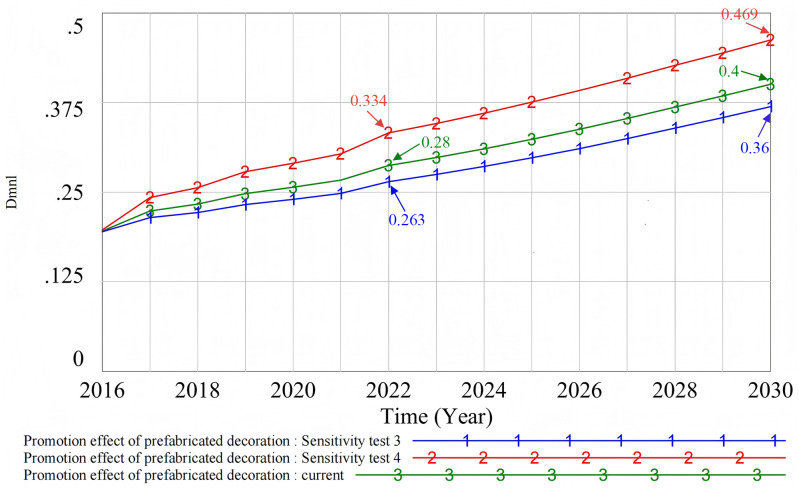
Sensitivity test of the number of incentive policies.

The sensitivity test of incentive policies (Fig 18) shows that, if government support is reduced (Sensitivity test 3), i.e., if the number of incentive policies is reduced, the promotion of prefabricated decoration will be reduced. On the contrary, if government support is strengthened (Sensitivity test 4), the promotion effect will be significantly improved. The introduction of new incentive policies can stimulate the willingness of developers and consumers to participate. This, in turn, promotes the wider application of prefabricated decoration and drives its marketisation process.

#### 4.3.2. Multivariable sensitivity test.

**Fig 19:**
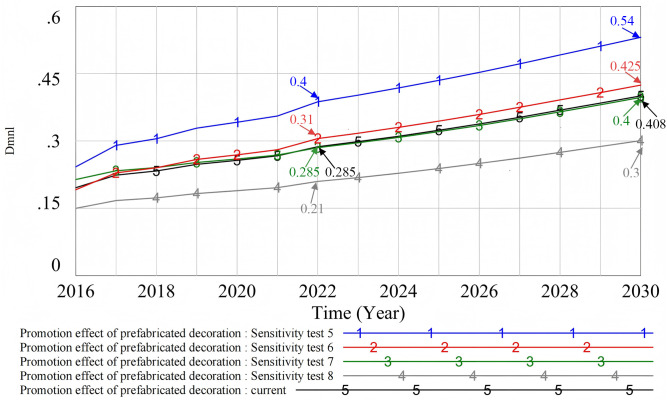
Multi-scenario sensitivity test.

In this paper, the price of prefabricated decoration and the number of incentive policies were taken as representatives of economic fluctuations and policy changes. A multivariate sensitivity analysis was carried out (see Fig 19) and four situational tests were designed: namely, low price and high incentive policy (Sensitivity test 5), high price and high incentive policy (Sensitivity test 6), low price and low incentive policy (Sensitivity test 7), and high price and low incentive policy (Sensitivity test 8). The analysis results show that, compared with the original state (Current), among the four policy combinations, the low-cost and high-incentive policy (Sensitivity test 5) had the most significant impact on promotion. Low cost significantly enhanced the willingness of developers and consumers to use prefabricated decoration, and the introduction of more incentive policies further promoted the popularization of prefabricated decoration. In contrast, the high-cost and low-incentive policy (Sensitivity test 8) had a negative impact on the promotion effect. It reduced the willingness of consumers and developers to choose prefabricated decoration and led them to prefer other decoration methods. Although the high-cost and high-incentive policy (Sensitivity test 6) was superior to the low-cost and low-incentive policy (Sensitivity test 7), its effect was not significant. In actual policy implementation, the government should choose the most suitable policy combination based on the economic environment and social needs. This can help promote the popularization of prefabricated decoration more effectively.

### 4.4. Countermeasures and suggestions

At present, the promotion of prefabricated decoration faces practical constraints, including limited policy support, low public acceptance, and weak transformation capacity. These issues limit its effectiveness in real-world applications. Therefore, combined with the simulation results, this study puts forward feasible and targeted countermeasures and suggestions from the perspective of government, developers and consumers.

#### 4.4.1. Government: accurate policy optimization incentives and standards.

The investment in prefabricated decoration is high in the early stage, and the local financial support ability is different. It is suggested that the central government set up guiding special funds, and local governments should support them, and implement differentiated subsidy mechanism according to the regional development stage and project nature (such as affordable housing and public rental housing). At the same time, the government can explore the establishment of an “prefabricated decoration incentive grading system”. Different incentive levels will be set based on the project's energy saving and emission reduction contributions, as well as the degree of standardization of parts. This will improve the efficiency of capital use. Vigorously promote the pilot project of prefabricated decoration. For construction projects developed by real estate enterprises, the prefabricate rate requirements will be clear at the land transfer stage. Developers who adopt the prefabricate-type decoration model will receive floor area ratio incentives or preferential loan interest rates. In addition, the government should promote the introduction of a technical standard system covering parts interfaces, installation processes and acceptance requirements, so as to provide institutional guarantee for enterprise investment decision-making and industrial chain coordination.

#### 4.4.2. Developers: differentiated digital transformation and supply optimization.

Considering the significant differences between developers in technical reserves and financial strength, it is suggested that large enterprises use BIM for collaborative design to improve the connection between prefabricated decoration and preliminary design. BIM’s application in virtual production and prefabricated can enable the integrated design of decoration, architecture, structure, and equipment. It can also more accurately carry out the arrangement of engineering quantity measurement and construction period plan, and realize the information management of the whole process. Small and medium-sized enterprises can reduce the technical threshold and procurement costs by joining the regional “prefabricated decoration cooperation network” to share design resources and the supply chain platform. To cope with price fluctuations of raw materials and parts, developers can sign a framework agreement with core suppliers. This agreement will set the price fluctuation range, performance conditions, and default compensation clauses, enhancing delivery stability. In addition, developers can enhance the competitiveness of prefabricated decoration by integrating the Internet. In the project construction stage, EPC mode is popularized. One of the characteristics of this mode is to design and construct at the same time. By integrating prefabricated decoration with the Internet, personalized decoration needs from different consumers are collected online. This allows decoration designers to intervene in advance and make targeted adjustments. It solves the problem of simplification brought by standardization and gives full play to the leading role of design in the whole engineering construction process. The use of internet tools has realized the de-intermediation and de-channelization of interior decoration, helping to reduce decoration costs.

#### 4.4.3. Consumers: Strengthen financial support and market confidence.

In view of consumers’ limited understanding of prefabricated decoration and heavy economic burden, the government can jointly set up a “green decoration loan channel” with commercial banks. This channel will provide low-interest or discount loans for buyers who choose prefabricated decoration products for the first time. Loan issuance can be tied to the list of officially certified projects to ensure transparent fund flow. To strengthen the trust mechanism, it is suggested to build a national unified “prefabricated decoration information disclosure platform”. This platform will release core information such as the source of project parts, the qualifications of construction units, and third-party quality inspection results, enhancing public supervision. At the same time, set up “prefabricated decoration experience demonstration sites” in key cities. Use the project marketing center's exhibition area to open mature prefabricated decoration cases to the public. This will help popularize the advantages of prefabricated decoration to consumers and increase their awareness and willingness to accept it.

## 5. Conclusions

The promotion of prefabricated decoration requires the cooperation of multiple stakeholders, with the government, developers, and consumers being the most critical. In this paper, 34 influencing factors were preliminarily screened through a literature review, a co-occurrence network of factors influencing the promotion of prefabricated decoration was constructed, and the key influencing factors were determined by calculating the centrality of factors. Using Vensim software, a dynamic model of the system was built. Through a multi-scenario simulation, we analyzed the effects of the key factors on the promotion of prefabricated decoration. Finally, according to the simulation results, some countermeasures and suggestions were put forward. Based on the above research, we draw the following conclusions:

First of all, there are many complicated factors involved in the promotion of prefabricated decoration, and different factors play different roles in the promotion system. Government support, the technical level, developers’ willingness to use, and consumers’ willingness to use are the four factors that have the greatest influence on the promotion of prefabricated decoration.

Secondly, developers and consumers are most sensitive to financial subsidies; thus, the government can appropriately increase financial subsidies to encourage developers to use prefabricated decoration and stimulate consumers’ willingness to go along with it. In addition to financial subsidies, the promulgation of mandatory policies is also an important measure to promote prefabricated decoration.

Finally, developers should increase the application of BIM technology over the whole life cycle of the project. This can effectively promote the coordination of the industrial chain and improve the promotion of prefabricated decoration. Consumer feedback and demands are very important for fine-tuning policies. They help the government to introduce more targeted measures, thus promoting the further development of the industry.

There were some limitations to this study:

(1)Many factors influence the promotion of prefabricated decoration, and the selected factors in this study may not be comprehensive. This may affect the research results to some extent. However, it is difficult to collect data as comprehensively as possible by manual work alone. Future research will explore combining deep learning with traditional system dynamics methods. In modeling large-scale complex systems, deep learning can support automated parameter tuning and pattern recognition to optimize the modeling process.

**(2)**This paper mainly uses the table function and lacks other simulation functions such as the logic function and test function. In a follow-up study, the above simulation functions could be used to simulate a sudden change, oscillation, or random interference of variables in the model[111] . Deep learning could also be used to analyze the influencing factors [112] to further mitigate the problem of complex data and multi-factor interaction. This would allow us to analyze changes in uncertain factors in prefabricated decoration promotion more comprehensively and make the whole simulation process more realistic.

## Supporting information

S1 Table Adjacency matrix. (XLSX)

S2 Table Attribute value of adjacency matrix. (XLSX)

S3 Table Equations used in the system dynamics model. (DOCX)

## References

[1] Cheng S, Zhou X, Zhang Y, Duan M, Gao J. Study on resilience factors and enhancement strategies in prefabricated building supply chains. Buildings. 2024;14(1):195. doi: 10.3390/buildings14010195

[2] Zhao Y, Liu L, Yu M. Comparison and analysis of carbon emissions of traditional, prefabricated, and green material buildings in materialization stage. J Cleaner Production. 2023;406:137152. doi: 10.1016/j.jclepro.2023.137152

[3] Zhang J, Xiang P, Zhong J, Zhang J, Wu Z, Antwi-Afari MF. Exploring Key Factors for Contractors in Opening Prefabrication Factories: A Chinese Case Study. Front Public Health. 2022;10. doi: 10.3389/fpubh.2022.837350 PMC8850311 35186853

[4] Hou YX, Li M, Li YF. Analysis on influencing factors of prefabricated decoration development based on ISM model. Building Economy. 2021;42(11):78–84.

[5] Weng YH, Li Y. Discussion on quality management of prefabricated interior decoration project. China Construction Decoration. 2024;155–157.

[6] Liu KJ, Wei ZF. Application and development of prefabricated construction technology in decoration engineering. Jushe. 2024;16:101–104.

[7] Xu M, Chang Y, Wei Y, Wang Y, Zhang P, Huang Z. Quantification and spatial pattern of embodied CO2 footprint of prefabricated buildings in urban agglomerations: A case study of Beijing–Tianjin–Hebei, China. Renewable Sustainable Energy Reviews. 2023;188:113837. doi: 10.1016/j.rser.2023.113837

[8] Shang Z, Wang F, Yang X. The Efficiency of the Chinese Prefabricated Building Industry and Its Influencing Factors: An Empirical Study. Sustainability. 2022;14(17):10695. doi: 10.3390/su141710695

[9] Cheng X, Liu Z. Analysis of Factors Affecting the Promotion of Prefabricated Interior Decoration Buildings in China Based on PEST-AHP. JES. 2022;14(3):201–207. doi: 10.3724/j.issn.1674-4969.22041301

[10] Deborah Aanuoluwa Soyombo. The role of policy and regulation in promoting green buildings. World J Adv Res Rev. 2024;22(1):139–150. doi: 10.30574/wjarr.2024.22.1.1047

[11] Hou YX, Yang S, Li YF, Zhou WZ. Study on quality control of prefabricated decoration construction based on DEMATEL-ISM model. Project Management Techniques. 2023;21:33–38.

[12] Kalamees T, Õiger K, Kõiv TA, Liias R, Kallavus U, Mikli L, et al. Technical condition of prefabricated concrete large panel apartment buildings in Estonia. In international conference on durability of building materials and components, Porto,Portugal. 2011;973–981.

[13] Zhao F. Analysis of influencing factors on quality management performance of prefabricated interior decoration in residential projects and improvement measures. Architecture and Budget. 2024;03:13–15.

[14] Smith R, Shilpa N. Prefabrication in developing countries: a case study of India. In: Wood Structures Symposium. 2009.

[15] Tam VWY, Fung IWH, Sing MCP, Ogunlana SO. Best practice of prefabrication implementation in the Hong Kong public and private sectors. J Cleaner Production. 2015;109:216–231. doi: 10.1016/j.jclepro.2014.09.045

[16] Navaratnam S, Ngo T, Gunawardena T, Henderson D. Performance Review of Prefabricated Building Systems and Future Research in Australia. Buildings. 2019;9(2):38. doi: 10.3390/buildings9020038

[17] Zhang X, Guo MJ, Zhang QY. The second exploration of foreign construction industrialization: the experience of American housing industrialization. Building. 2018;05:54–57.

[18] Zhang X, Liang JS, Zhang QY. The fourth exploration of foreign building industrialization Sweden: promoting sustainable housing industrialization. Building. 2018;08:48–50.

[19] Kirschke P, Sietko D. The Function and Potential of Innovative Reinforced Concrete Prefabrication Technologies in Achieving Residential Construction Goals in Germany and Poland. Buildings. 2021;11(11):533. doi: 10.3390/buildings11110533

[20] Abd Jalil A, Jaafar M, Md Fazil F, Nawi N, Shazwan Hashim MA. Why Current Procurement Systems Require Modifications to Suit the Natures of Malaysian Pre-fabricated Construction. Green Infrastructure. Springer Nature Singapore. 2023；139–159. doi: 10.1007/978-981-99-7003-2_8

[21] Xu Z, Zayed T, Niu Y. Comparative analysis of modular construction practices in mainland China, Hong Kong and Singapore. J Cleaner Production. 2020;245:118861. doi: 10.1016/j.jclepro.2019.118861

[22] Guiding opinions of General Office of the State Council on vigorously developing prefabricated buildings: Guo Ban Fa [2016] No.71. 2016. https://www.gov.cn/zhengce/content/2016-09/30/content_5114118.htm#:~:text=%E7%BB%93%E5%90%88%E8%8A%82%E8%83%BD%E5%87%8F%E6%8E%92

[23] Wang YP. Thinking on prefabricated decoration design based on green and low carbon concept. Jushe. 2024;06:80–82.

[24] He B. On the application and development of prefabricated decoration. Mass Standardization. 2024;07:125–127.

[25] Hangzhou prefabricated decoration pilot work implementation plan. Concrete. 2023;06:187.

[26] Jiangxi Provincial Department of Housing and Urban-Rural Development. Guiding Opinions on Strengthening the Construction Management of Fully Decorated Finished Houses: J.Z. No.10. 2021. https://www.jiangxi.gov.cn/art/2021/12/30/art_398_3812596.html#:~:text=%E8%BF%91%E6%97%A5%EF%BC%8C%E7%9C%81%E4%BD%8F%E6%88%BF

[27] Zhang J, Xiang P, Zhong J, Zhang J, Wu Z, Antwi-Afari MF. Exploring Key Factors for Contractors in Opening Prefabrication Factories: A Chinese Case Study. Front Public Health. 2022;10:837350. doi: 10.3389/fpubh.2022.837350 35186853 PMC8850311

[28] Lu W, Chen K, Xue F, Pan W. Searching for an optimal level of prefabrication in construction: An analytical framework. J Cleaner Production. 2018;201:236–245. doi: 10.1016/j.jclepro.2018.07.319

[29] Wu G, Yang R, Li L, Bi X, Liu B, Li S, et al. Factors influencing the application of prefabricated construction in China: From perspectives of technology promotion and cleaner production. J Cleaner Production. 2019;219:753–762. doi: 10.1016/j.jclepro.2019.02.110

[30] Jayawardana J, Sandanayake M, Jayasinghe S, Kulatunga A, Zhang G. Key barriers and mitigation strategies towards sustainable prefabricated construction – a case of developing economies. Engineering, Construction and Architectural Management. 2024;32(7):4796–4833. doi: 10.1108/ecam-09-2023-0978

[31] Li Z, Zhang S, Meng Q, Hu X. Barriers to the development of prefabricated buildings in China: a news coverage analysis. ECAM. 2020;28(10):2884–2903. doi: 10.1108/ecam-03-2020-0195

[32] Zhong C, Zhang M, Cui X, Liu Z. Comprehensive Evaluation of China's Prefabricated Decoration Cost Based on Analytic Hierarchy Process. Advances in Civil Engineering. 2020;2020(1). doi: 10.1155/2020/1583748

[33] Jiang L, Li Z, Li L, Gao Y. Constraints on the Promotion of Prefabricated Construction in China. Sustainability. 2018;10(7):2516. doi: 10.3390/su10072516

[34] Ouyang J. Intelligent construction promotes the development of prefabricated buildings. Housing and Real Estate. 2023;23:40–41.

[35] Zeng SL, Zhang DL, Liu YM, Tan ZJ, Huang Q. Study on standardization of product selection for prefabricated building decoration. China Construction Decoration. 2023;15:97–99.

[36] Zhang YC, Cao XD, Zhang LZ, Xu Q, Qi SW. Analysis on the implementation mode of integrated full decoration of prefabricated residential buildings. Gansu Science and Technology. 2022;38(12):8–11.

[37] Cai J, Yang TT, Yang Y, Gao B. Research on the constraints of the development of prefabricated buildings in Hangzhou based on ISM-MICMAC. J Hubei University of Technology. 2024;39(3):46–51.

[38] Belton V, Stewart TJ. Multiple Criteria Decision Analysis. Springer US. 2002. doi: 10.1007/978-1-4615-1495-4

[39] Liang R, Li R, Yan X, Xue Z, Wei X. Evaluating and selecting the supplier in prefabricated megaprojects using extended fuzzy TOPSIS under hesitant environment: a case study from China. ECAM. 2022;30(5):1902–31. doi: 10.1108/ecam-09-2021-0793

[40] Wang Q, Wang Z. TOPSIS Method Based on Prospect Theory for Interval Value Hesitant Fuzzy Set MAGDM and Its Application to Digital Economy. In: Proceedings of the 2023 6th International Conference on Big Data Technologies. 2023；319–325. 10.1145/3627377.3627427

[41] Zhao H, Wang S, Lu C. A study on site selection of wind power plant based on prospect theory and VIKOR: a case study in China. K. 2024;54(7):4043–4066. doi: 10.1108/k-01-2024-0022

[42] Li Y, Cai Q, Wei G. PT-TOPSIS methods for multi-attribute group decision making under single-valued neutrosophic sets. KES. 2023;27(2):149–166. doi: 10.3233/kes-230039

[43] Sánchez-Garrido AJ, Navarro IJ, Yepes V. Multi-criteria decision-making applied to the sustainability of building structures based on Modern Methods of Construction. J Cleaner Production. 2022;330:129724. doi: 10.1016/j.jclepro.2021.129724

[44] Shahpari M, Saradj FM, Pishvaee MS, Piri S. Assessing the productivity of prefabricated and in-situ construction systems using hybrid multi-criteria decision making method. J Building Engineering. 2020;27:100979. doi: 10.1016/j.jobe.2019.100979

[45] Hassan Ali A, Farouk Kineber A, Elshaboury N, Arashpour M, Osama Daoud A. Analysing multifaceted barriers to modular construction in sustainable building projects: a comprehensive evaluation using multi-criteria decision making. Int J Construction Management. 2024;25(1):1–17. doi: 10.1080/15623599.2023.2299557

[46] Chang L, Nordin N, Zhao S, Gu X, Zhao Y. TOPSIS prefabricated building construction evaluation based on interval-valued Pythagorean fuzzy numbers based on prospect theory. Sci Rep. 2025;15(1). doi: 10.1038/s41598-025-85729-1 PMC11758387 39848985

[47] Assaf M, Hussein M, Abdelkhalek S, Zayed T. A Multi-Criteria Decision-Making Model for Selecting the Best Project Delivery Systems for Offsite Construction Projects. Buildings. 2023;13(2):571. doi: 10.3390/buildings13020571

[48] Ighravwe DE, Oke SA. A multi-criteria decision-making framework for selecting a suitable maintenance strategy for public buildings using sustainability criteria. J Building Engineering. 2019;24:100753. doi: 10.1016/j.jobe.2019.100753

[49] Yuan Z, Man Q, Guan Z, Yi C, Zheng M, Chang Y, et al. Simulation and optimization of prefabricated building construction considering multiple objectives and uncertain factors. J Building Eng. 2024;86:108830. doi: 10.1016/j.jobe.2024.108830

[50] Wu W, Sheng L, Tang F, Zhang A, Liu J. A system dynamics model of green innovation and policy simulation with an application in Chinese manufacturing industry. Sustainable Prod Consumption. 2021;28:987–1005. doi: 10.1016/j.spc.2021.07.007

[51] Cheng B, Wei Y, Zhang W, Zhou X, Chen H, Huang L, et al. Evolutionary Game Simulation on Government Incentive Strategies of Prefabricated Construction: A System Dynamics Approach. Complexity. 2020;2020:1–11. doi: 10.1155/2020/8861146

[52] Li Z, Zhang S, Meng Q. Modeling Adoption Behavior of Prefabricated Building with Multiagent Interaction: System Dynamics Analysis Based on Data of Jiangsu Province. Computational Intelligence and Neuroscience. 2021;2021(1). doi: 10.1155/2021/3652706 PMC8651351 34887914

[53] Chen J, Liu P, Lin B, Zhou H, Papachristos G. The diffusion of prefabrication technology and its potential for CO2 emissions reduction in China: A combined system dynamics and agent-based study. Technological Forecasting and Social Change. 2025;210:123890. doi: 10.1016/j.techfore.2024.123890

[54] Li CZ, Hong J, Fan C, Xu X, Shen GQ. Schedule delay analysis of prefabricated housing production: A hybrid dynamic approach. J Cleaner Prod. 2018;195:1533–1545. doi: 10.1016/j.jclepro.2017.09.066

[55] Gao Y, Tian X-L. Prefabrication policies and the performance of construction industry in China. J Cleaner Prod. 2020;253:120042. doi: 10.1016/j.jclepro.2020.120042

[56] Han Y, Mtisi RS, Zhou J. Analyzing the influencing factors for contractors in opening prefabrication factories: a Sub-Saharan African case study. ECAM. 2025. doi: 10.1108/ecam-08-2024-1026

[57] Xie L, Chen Y, Xia B, Hua C. Importance‐Performance Analysis of Prefabricated Building Sustainability: A Case Study of Guangzhou. Ad Civil Eng. 2020;2020(1). doi: 10.1155/2020/8839118

[58] Wang D-Y, Wang X. Supply Chain Consequences of Government Subsidies for Promoting Prefabricated Construction and Emissions Abatement. J Manage Eng. 2023;39(5). doi: 10.1061/jmenea.meeng-5285

[59] Wang S, Wang C, Li W, Zhao D. Study on the operational efficiency of prefabricated building industry bases in Western China based on the DEA model. Arab J Geosci. 2021;14(6). doi: 10.1007/s12517-021-06798-w

[60] Shi Q, Wang Z, Zhu J. Developing Collaborative Driving Mechanism of Prefabricated Buildings Using Multiagent Stochastic Evolutionary Game. J Constr Eng Manage. 2024;150(6). doi: 10.1061/jcemd4.coeng-14396

[61] Wang Y, Wang F, Sang P, Song H. Analysing factors affecting developers’ behaviour towards the adoption of prefabricated buildings in China. Environ Dev Sustain. 2021;23(10):14245–14263. doi: 10.1007/s10668-021-01265-8

[62] Jiang W, Luo L, Wu Z, Fei J, Antwi-Afari MF, Yu T. An Investigation of the Effectiveness of Prefabrication Incentive Policies in China. Sustainability. 2019;11(19):5149. doi: 10.3390/su11195149

[63] Tan Q, Ye M. Allocation of Environmental Responsibilities in the Prefabricated Construction Supply Chain: Exploring the Influence of Government Subsidies. J Constr Eng Manage. 2024;150(11). doi: 10.1061/jcemd4.coeng-15018

[64] Ren X, Jia C, Wang M. Policy Effect on Technology Innovation in Prefabricated Buildings: An Empirical Study Using the Difference-in-Differences Approach. J Constr Eng Manage. 2024;150(10). doi: 10.1061/jcemd4.coeng-14524

[65] Han YH, Fang X, Zhao XY, Wang LF. Exploring the impact of incentive policy on the development of prefabricated buildings: A scenario-based system dynamics model. Engineering, Construction and Architectural Management. 2024;31(12):4697–4725. Available from: doi: 10.1108/ECAM-01-2023-0084

[66] Luo T, Xue X, Wang Y, Xue W, Tan Y. A systematic overview of prefabricated construction policies in China. Journal of Cleaner Production. 2021;280:124371. doi: 10.1016/j.jclepro.2020.124371

[67] Jiang W, Qi X. Pricing and assembly rate decisions for a prefabricated construction supply chain under subsidy policies. PLoS ONE. 2022;17(1):e0261896. doi: 10.1371/journal.pone.0261896 PMC8735663 34990462

[68] Cao X, Li X, Zhu Y, Zhang Z. A comparative study of environmental performance between prefabricated and traditional residential buildings in China. Journal of Cleaner Production. 2015;109:131–143. doi: 10.1016/j.jclepro.2015.04.120

[69] Jaillon L, Poon CS. The evolution of prefabricated residential building systems in Hong Kong: A review of the public and the private sector. Automation in Construction. 2009;18(3):239–248. doi: 10.1016/j.autcon.2008.09.002

[70] Jaillon L, Poon CS. The evolution of prefabricated residential building systems in Hong Kong: A review of the public and the private sector. Automation in Construction. 2009;18(3):239–248. doi: 10.1016/j.autcon.2008.09.002

[71] Xu S, Zhou L, Zou PXW. What influences stakeholders’ decision in adopting blockchain-based quality tracking systems in prefabricated construction. Engineering, Construction and Architectural Management. 2024;31(6):2224–2247. Available from: doi: 10.1108/ECAM-06-2022-0501

[72] Xia M, Zhao L, Qiao Y, Yuan Z, Cui Y, Zhao L, et al. Analysis of Factors Affecting the Quality of Precast Components Based on Structural Equation Modeling. Arab J Sci Eng. 2021;47(4):4171–4185. doi: 10.1007/s13369-021-05991-z

[73] Liang H, Zhang S, Su Y. Evaluating the Efficiency of Industrialization Process in Prefabricated Residential Buildings Using a Fuzzy Multicriteria Decision‐Making Method. Mathematical Problems in Engineering. 2017;2017(1). doi: 10.1155/2017/6078490

[74] Ji Y, Qi K, Qi Y, Li Y, Li HX, Lei Z, et al. BIM-based life-cycle environmental assessment of prefabricated buildings. ECAM. 2020;27(8):1703–1725. doi: 10.1108/ecam-01-2020-0017

[75] Gan X, Liu L, Wen T. Evaluation of policies on the development of prefabricated construction in china: an importance-performance analysis. J Green Building. 2022;17(1):149–168. doi: 10.3992/jgb.17.1.147

[76] Du Q, Pang Q, Bao T, Guo X, Deng Y. Critical factors influencing carbon emissions of prefabricated building supply chains in China. J Cleaner Prod. 2021;280:124398. doi: 10.1016/j.jclepro.2020.124398

[77] Sun P, Zhang N, Zuo J, Mao R, Gao X, Duan H. Characterizing the generation and flows of building interior decoration and renovation waste: A case study in Shenzhen City. J Cleaner Production. 2020;260:121077. doi: 10.1016/j.jclepro.2020.121077

[78] Gong R, Qi Y, Lian C, Gao X, Yao F, Srinivasan V, et al. Developing poplar wood into a green structure-decoration integrated material for prefabricated wooden building application. J Building Eng. 2024;90:109386. doi: 10.1016/j.jobe.2024.109386

[79] Wang X, Tang Q. Research on detailed design methods of precast components in prefabricated buildings based on BIM technology. Heliyon. 2024;10(16):e35922. doi: 10.1016/j.heliyon.2024.e35922 PMC11363855 39220974

[80] Gong C, Xu H, Xiong F, Zuo J, Dong N. Factors impacting BIM application in prefabricated buildings in China with DEMATEL-ISM. CI. 2021;23(1):19–37. doi: 10.1108/ci-06-2021-0115

[81] Tavares V, Soares N, Raposo N, Marques P, Freire F. Prefabricated versus conventional construction: Comparing life-cycle impacts of alternative structural materials. J Building Engineering. 2021;41:102705. doi: 10.1016/j.jobe.2021.102705

[82] Hu R, Chen K, Fang W, Zheng L, Xu J. The technology-environment relationship revisited: Evidence from the impact of prefabrication on reducing construction waste. J Cleaner Production. 2022;341:130883. doi: 10.1016/j.jclepro.2022.130883

[83] Lu W, Lee WMW, Xue F, Xu J. Revisiting the effects of prefabrication on construction waste minimization: A quantitative study using bigger data. Resources, Conservation and Recycling. 2021;170:105579. doi: 10.1016/j.resconrec.2021.105579

[84] Zhang S, Yuan M, Li L. Exploring the Impact Mechanism of Interface Management of Prefabricated Construction Projects. Sustainability. 2022;14(21):14440. doi: 10.3390/su142114440

[85] Wu Z, Li S, Lin Y, Luo L, Xue H, Fordjour Antwi-Afari M. Analysis of factors affecting the prefabricated housing promotion from the perspective of stakeholders. Energy and Buildings. 2024;320:114588. doi: 10.1016/j.enbuild.2024.114588

[86] Han Y, Xu X, Zhao Y, Wang X, Chen Z, Liu J. Impact of consumer preference on the decision-making of prefabricated building developers. J Civil Eng Manag. 2022;28(3):166–176. doi: 10.3846/jcem.2022.15777

[87] Zhou Z, Shen G, Xue J, Sun C, Liu Y, Cong W, et al. The formation of citizens’ intentions to purchase prefabricated housing in China: the integrating theory of planned behavior and norm activation model. ECAM. 2023;32(3):1759–1780. doi: 10.1108/ecam-05-2023-0473

[88] Zhou Z, Wei C, Shen GQ, Xue J, Liu Y, Wang Y, et al. Citizens’ acceptance of prefabricated housing in China: the role of procedural justice and uncertainty. Engineering, Construction and Architectural Management. 2024;ahead-of-print(ahead-of-print). Available from: 10.1108/ECAM-01-2024-0041

[89] Onyszkiewicz J, Sadowski K. Proposals for the revitalization of prefabricated building facades in terms of the principles of sustainable development and social participation. J Building Engineering. 2022;46:103713. doi: 10.1016/j.jobe.2021.103713

[90] Han Y, Wang L, Kang R. Influence of consumer preference and government subsidy on prefabricated building developer's decision-making: a three-stage game model. J Civil Eng Manag. 2023;29(1):35–49. doi: 10.3846/jcem.2023.18038

[91] Zhang K, Tsai J-S. Identification of Critical Factors Influencing Prefabricated Construction Quality and Their Mutual Relationship. Sustainability. 2021;13(19):11081. doi: 10.3390/su131911081

[92] Luo L, Qiping Shen G, Xu G, Liu Y, Wang Y. Stakeholder-Associated Supply Chain Risks and Their Interactions in a Prefabricated Building Project in Hong Kong. J Manage Eng. 2019;35(2). doi: 10.1061/(asce)me.1943-5479.0000675

[93] Yang RJ, Zou PXW. Stakeholder-associated risks and their interactions in complex green building projects: A social network model. Building and Environment. 2014;73:208–222. doi: 10.1016/j.buildenv.2013.12.014

[94] Yu T, Shen GQ, Shi Q, Lai X, Li CZ, Xu K. Managing social risks at the housing demolition stage of urban redevelopment projects: A stakeholder-oriented study using social network analysis. International Journal of Project Management. 2017;35(6):925–941. doi: 10.1016/j.ijproman.2017.04.004

[95] Kuai P, Li W, Cheng R, Cheng G. An application of system dynamics for evaluating planning alternatives to guide a green industrial transformation in a resource-based city. Journal of Cleaner Production. 2015;104:403–412. doi: 10.1016/j.jclepro.2015.05.042

[96] Kwak S. Policy analysis of Hanford tank farm operations with system dynamics approach. Boston: MIT. 1996.

[97] Guo D, Li G, Hu N, Hou J. System Dynamics Analysis of Man-Machine Efficacy in Plateau Mines. IEEE Access. 2021;9:18072–18084. doi: 10.1109/access.2021.3052211

[98] Xu X, Zhang W, Li N, Xu H. A bi-level programming model of resource matching for collaborative logistics network in supply uncertainty environment. Journal of the Franklin Institute. 2015;352(9):3873–3884. doi: 10.1016/j.jfranklin.2015.01.021

[99] Zhu X, Liu F. Research on Behavior Model of Rumor Maker Based on System Dynamics. Complexity. 2017;2017:1–9. doi: 10.1155/2017/5094218

[100] Du Q, Bao T, Li Y, Huang Y, Shao L. Impact of prefabrication technology on the cradle-to-site CO2 emissions of residential buildings. Clean Techn Environ Policy. 2019;21(7):1499–1514. doi: 10.1007/s10098-019-01723-y

[101] Nguyen T, Cook S, Ireland V. Application of System Dynamics to Evaluate the Social and Economic Benefits of Infrastructure Projects. Systems. 2017;5(2):29. doi: 10.3390/systems5020029

[102] Du Q, Yang M, Wang Y, Wang X, Dong Y. Dynamic simulation for carbon emission reduction effects of the prefabricated building supply chain under environmental policies. Sustainable Cities and Society. 2024;100:105027. doi: 10.1016/j.scs.2023.105027

[103] Barlas Y. Formal aspects of model validity and validation in system dynamics. Syst Dyn Rev. 1996;12(3):183–210. doi: 10.1002/(sici)1099-1727(199623)12:3<183::aid-sdr103>3.0.co;2-4

[104] Zhang W, Zhang M, Wu S, Liu F. A complex path model for low-carbon sustainable development of enterprise based on system dynamics. Journal of Cleaner Production. 2021;321:128934. doi: 10.1016/j.jclepro.2021.128934

[105] Guo F, Wang J, Song Y. How to promote sustainable development of construction and demolition waste recycling systems: Production subsidies or consumption subsidies?. Sustainable Production and Consumption. 2022;32:407–423. doi: 10.1016/j.spc.2022.05.002

[106] Liu C, Yang Y, Zhao X, Xu X, Hao JL, Ma W. Reducing carbon emissions by using prefabricated decoration floor systems. J Green Building. 2023;18(1):119–145. doi: 10.3992/jgb.18.1.119

[107] Wu ZZ, Yang KJ, Wu ZM, Xue H, Li SH, Antwi-Afari MF. Investigating the mechanism of developers’ willingness to adopt prefabricated housing using an integrated DEMATEL-SD framework. Engineering, Construction and Architectural Management. 2024;31:2392–2414. Available from: doi: 10.1108/ECAM-05-2022-0422

[108] Liu H, Sydora C, Altaf MS, Han S, Al-Hussein M. Towards sustainable construction: BIM-enabled design and planning of roof sheathing installation for prefabricated buildings. J Cleaner Production. 2019;235:1189–1201. doi: 10.1016/j.jclepro.2019.07.055

[109] Pan HZ, Yang BF, Pan YW, Luo ZH. Evolutionary game of incentive strategy for Chinese prefabricated buildings based on system dynamics from the perspective of prospect theory. Engineering, Construction and Architectural Management 2024. Available from: doi: 10.1108/ECAM-10-2023-1031

[110] Song Y, Wang J, Liu D, Huangfu Y, Guo F, Liu Y. The Influence of Government's Economic Management Strategies on the Prefabricated Buildings Promoting Policies: Analysis of Quadripartite Evolutionary Game. Buildings. 2021;11(10):444. doi: 10.3390/buildings11100444

[111] Xue X, Zhang X, Wang L, Skitmore M, Wang Q. Analyzing collaborative relationships among industrialized construction technology innovation organizations: A combined SNA and SEM approach. Journal of Cleaner Production. 2018;173:265–277. doi: 10.1016/j.jclepro.2017.01.009

[112] Ruan Y. The Cultural Value Validity of Digital Media Art Based on Deep Learning Network Model. Advances in Multimedia. 2022;2022:1–11. doi: 10.1155/2022/3799350

