# Supplementary material for: A System dynamics analysis of the factors influencing the promotion of prefabricated decoration, based on a co-occurrence network
Source: PLoS One. 2025 Sep 4;20(9):e0331703. doi: 10.1371/journal.pone.0331703 (PMC12410792; doi:10.1371/journal.pone.0331703)
Supplement: S3 Table — (DOCX) [file pone.0331703.s003.docx]

| **Table S3. Equations used in the system dynamics model.** | | | |
| --- | --- | --- | --- |
| **Variable** | **Variable name** | **Type** | **Model equation** |
| G1 | Government support | State  variable | INTEG (Government support strength,0) |
| G6 | Hebei Province GDP total amount |  | INTEG (28474.12,GDP growth) |
| G7 | Hebei Province’s total population |  | INTEG (7360.15,Population increase) |
| C3 | Total market demand |  | INTRG (Demand increase,15) |
| D1 | Technical level |  | INTEG (Technical level growth rate,0.145) |
| D6 | Total market supply |  | INTEG (Supply increase,20) |
| — | Government support strength | Rate  variable | Sustainable development appeal*0.683+Amount of waste generated*0.317 |
| _ | GDP growth rate |  | With LOOKUP (Time, ([(2016,0) - (2030,1)], (2016,0.067), (2017,0.066), (2018,0.065), (2019,0.067), (2020,0.038), (2021,0.065), (2022,0.038), (2025,0.032), (2028,0.03), (2030,0.027))) |
| _ | Population increase |  | Hebei Province’s total population  *Population growth rate |
| — | Technical level growth rate |  | 0.334*Professional and technical talent level+0.245*Enterprise management model+0.421*Scope of BIM technology |
| — | Demand increase |  | Per capita housing area*Population increase*0.179 |
| G2 | Government propaganda | Auxiliary  variable | Government support*0.19+Sustainable development appeal*0.81 |
| G3 | Government supervision |  | Policy support*0.354+Sustainable development appeal*0.646 |
| G8 | Per capita GDP |  | Hebei Province GDP total amount*10000/Hebei Province’s total population |
| G9 | Per capita disposable income |  | Per capita GDP*Consumption ratio |
| S2 | Decoration price income ratio |  | Decoration price/Per capita disposable income |
| D2 | Standardization of components |  | Technical level*0.125 |
| D3 | Degree of improvement in technical standards |  | 0.37*Standardization of universal interfaces+0.4*Equipment pipeline is easy to disassemble+0.23*Standardization of components |
| D7 | New area of prefabricated decoration |  | Total market supply*0.413 |
| D8 | Decoration efficiency |  | Scope of BIM technology*0.56 |
| D9 | Integrity of the industrial chain |  | 0.58*Participants in collaboration+0.42*The degree of improvement of technical standards |
| D11 | Enterprise decoration experience |  | New area of prefabricated decoration*0.32 |
| D12 | Use of recycled materials |  | 0.37*Sustainable development appeal+0.63*Technical level |
| D13 | Amount of waste generated |  | Use of recycled materials*0.3 |
| D14 | Participants in collaboration |  | Industry standards*0.4 |
| D15 | Developers’ willingness to use |  | 0.081*Industry standards+  0.23*Government Support+0.252*Consumers’ willingness to use+0.05*Incentive policy+0.11*Industry development plan+  0.082*Decoration efficiency+  0.168*Financial subsidy |
| C1 | Consumers’ willingness to use |  | 0.13*Government propaganda+0.07*Government supervision+0.26*Material quality and acceptance effect+0.23*Incentive policy+0.15*Decoration price income ratio+0.26*Financial subsidy |
| C2 | Sustainable development appeal |  | WITH LOOKUP (Time, [(2016,0) - (2030,1)], (2016,0.003), (2017,0.12), (2018,0.15), (2019,0.2), (2020,0.22), (2021,0.24), (2022,0.3), (2025,0.34), (2028,0.38), (2030,0.4))) |
| M1 | Material quality and acceptance |  | Technical level*0.424 |
| M2 | Standardization of universal interfaces |  | Technical level*0.256 |
| M3 | Equipment pipeline easy to disassemble |  | Technical level*0.167 |
| G4 | Incentive policy | Constant variable | 0/1/5 |
| G5 | Financial subsidy |  | 100 |
| _ | Consumption ratio |  | 0.18 |
| I1 | Industry standards |  | 0.35 |
| I2 | Industry development plan |  | 0.25 |
| S1 | Decoration price |  | 2250 |
| D4 | Scope of BIM technology |  | 0.537 |
| D5 | Professional and technical talent level |  | 0.355 |
| — | Per capita housing area |  | 60 |
| D10 | Enterprise management model |  | 0/1 |

*The data comes from literature, questionnaires and Hebei Provincial Bureau of Statistics.
